# Toxicity and possible mechanisms of action of honokiol from *Magnolia denudata* seeds against four mosquito species

**DOI:** 10.1038/s41598-018-36558-y

**Published:** 2019-01-23

**Authors:** Zhangqian Wang, Haribalan Perumalsamy, Xue Wang, Young-Joon Ahn

**Affiliations:** 1 Department of Agricultural Biotechnology, Seoul National University, Seoul, 08826 Republic of Korea; 2 Key Laboratory of Combinatorial Biosynthesis and Drug Discovery (Wuhan University), Ministry of Education, and Wuhan University School of Pharmaceutical Sciences, Wuhan, 430071 Hubei China; 3 Research Institute of Agriculture and Life Sciences, Seoul National University, Seoul, 08826 Republic of Korea; 4 School of Pharmaceutical Science, Wenzhou Medical University, Wenzhou, 325035 Zhejiang China

## Abstract

This study was performed to determine the toxicity and possible mechanism of the larvicidal action of honokiol, extracted from *Magnolia denudata* seeds, and its 10 related compounds against third-instar larvae of insecticide-susceptible *Culex pipiens pallens*, *Aedes aegypti*, and *Aedes albopictus* and *Anopheles sinensis* resistant to deltamethrin and temephos. Honokiol (LC_50_, 6.13–7.37 mg/L) was highly effective against larvae of all of the four mosquito species, although the toxicity of the compound was lower than that of the synthetic larvicide temephos. Structure–activity relationship analyses indicated that electron donor and/or bulky groups at the *ortho* or *para* positions of the phenol were required for toxicity. Honokiol moderately inhibited acetylcholinesterase and caused a considerable increase in cyclic AMP levels, indicating that it might act on both acetylcholinesterase and octopaminergic receptors. Microscopy analysis clearly indicated that honokiol was mainly targeted to the midgut epithelium and anal gills, resulting in variably dramatic degenerative responses of the midgut through sequential epithelial disorganization. Honokiol did not affect the AeCS1 mRNA expression level in *Ae*. *aegypti* larvae, but did enhance expression of the genes encoding vacuolar-type H^+^-ATPase and aquaporin 4, indicating that it may disturb the Na^+^, Cl^−^ and K^+^ co-transport systems. These results demonstrate that honokiol merits further study as a potential larvicide, with a specific target site, and as a lead molecule for the control of mosquito populations.

## Introduction

The yellow fever mosquito *Aedes aegypti* and Asian tiger mosquito *Aedes albopictus* are both found in tropical and subtropical regions around the world^1^; by contrast, the common Oriental mosquito *Anopheles sinensis* and common house mosquito *Culex pipiens pallens* are found in South East Asia^2^. Various diseases caused by these mosquito species represent several of the most serious global public health problems^1–4^. The prevalence and severity of these clinical conditions are distinctly increasing and closely associated with global warming, tainted fresh water pools, and increased international travel^5–7^. An estimated 198 million cases of malaria occurred globally in 2013 with at least 584,000 deaths, primarily among children under 5 years of age^8^. A recent study determined that almost 4 billion people are at risk of infection with dengue viruses in 128 countries worldwide^9,10^. Estimates of the number of annual symptomatic dengue infections range from 50 million to 100 million^10,11^, including approximately 10,000–20,000 deaths annually, primarily among children^11,12^. From 1999 to 2015, 43,937 West Nile virus disease cases (including 20,265 neuroinvasive disease cases) were reported in the United States (US), resulting in 1,911 deaths^13^. Widespread insecticide resistance^14^ has been one of the major obstacles to cost-effective integrated mosquito management (IMM) programs. In addition, the number of approved insecticides will probably be reduced soon in the US^15^ and the European Union^16^ as reregistration occurs. The removal of conventional mosquitocidal products from markets due to increases in insecticide resistance or other concerns will seriously affect the proliferation of mosquitoes. Therefore, there is a pressing need to develop new improved mosquito control alternatives with novel target sites to establish a biorational management strategy and tactics because vaccines have limited effectiveness in controlling malaria^17^ and dengue^18^.

Plants may provide potential sources of mosquito control products largely because they are sources of bioactive secondary metabolites (SMs) that are perceived by the general public to be relatively safe and pose less risk to the environment as well as to have minimal impact on human and animal health^19–22^. SMs act at multiple, novel target sites^20–24^, thereby reducing the potential for resistance^25,26^. Efforts to evaluate these benefits of botanical insecticides have resulted in numerous papers being published annually^27^. However, the mechanisms of their larvicidal action have not been clearly defined or understood. Histopathological studies have revealed that the midgut of insects is one of the main target organs for many xenobiotics, including SMs^28–30^. Previous studies have shown that a hydrodistillate from the seeds of the yulan magnolia (lily tree) plant *Magnolia denudata* Desr. (Magnoliales: Magnoliaceae) exhibited potent toxicity against third-instar *Cx*. *pipiens pallens* and *Ae*. *aegypti* larvae^31^. Approximately 230 species of the genus *Magnolia*, which is one of the most primitive angiosperms, are widely distributed throughout the temperate Northern Hemisphere^32^. Approximately two-thirds of this species are currently distributed in temperate and tropical regions of eastern and southeastern Asia. This plant species contains a variety of SMs, such as alkaloids, lignans, neolignans, phenylpropanoids, and terpenoids^33,34^. *Magnolia denudata* is native to eastern and southern China, and its dried flower buds have been used for the treatment of emphysema, nasal congestion, sinusitis, and allergic rhinitis^33,34^. However, no previous studies have investigated the potential use of *M*. *denudata* for managing mosquitoes, particularly insecticide-resistant mosquitoes, for future commercialization, as well as the mechanisms of larvicidal action of the plant constituents. Traditional and folk medicinal uses of *Magnolia* species have been well-documented by Kelm and Nair^33^ and Li *et al*.^34^. Sukumar *et al*.^19^ noted that the most promising botanical anti-mosquito products are plants in the families Asteraceae, Cladophoraceae, Lamiaceae, Meliaceae, Oocystaceae, and Rutaceae, although the anti-mosquito activity can vary significantly according to plant species, chemotypes, plant tissue type, plant age, geographic conditions, solvent used for extraction, and mosquito species.

The aim of this study was to assess whether the neolignan honokiol; two fatty acids, linoleic acid and palmitic acid, that were extracted from *M*. *denudata* seeds; and magnolol, a structural isomer of honokiol, showed contact toxicity against third-instar larvae of insecticide-susceptible laboratory colonies of *Cx*. *pipiens pallens*, *Ae*. *aegypti*, and *Ae*. *albopictus* and laboratory colonies of *An*. *sinensis* resistant to deltamethrin and temephos. The results were compared with those of the organophosphate insecticide (OP) temephos, which is a currently used mosquito larvicide, to assess their potential for use as future commercial mosquito larvicides. Quantitative structure–activity relationship (QSAR) analysis of honokiol and 10 of its structurally-related phenolic compounds is discussed. The morphological changes in the midgut and anal gills of larval *Ae*. *aegypti* were also examined using light microscopy and transmission electron microscopy (TEM). In addition, gene expression analyses of larval *Ae*. *aegypti* chitin synthase (AeCS), vacuolar-type H^+^-ATPase (AaV-type H^+^-ATPase), and aquaporin (AQP) 4 (AaAQP4), which acts as a water channel in anal gills, after treatment with honokiol and magnolol were used to determine the possible target sites of the two neolignans. Finally, the possible mechanism underlying the larvicidal actions of the two neolignans against *Ae*. *aegypti* was elucidated using biochemical, histologic, and molecular analyses.

## Results

### Contact mortality bioassay-guided fractionation and identification

The fractions obtained from solvent partitioning of the methanol extract of *M*. *denudata* seeds were tested for larvicidal activity against third-instar larvae of insecticide-susceptible *Cx*. *pipiens pallens* and *Ae*. *aegypti* (Table 1). Significant differences in toxicity were observed among the fractions and were used to identify the peak activity fractions for the next step in purification. As judged by the 24 h LC_50_ values, the hexane-soluble fraction was the most potent larvicide, whereas no toxicity was obtained using the chloroform-, ethyl acetate-, butanol-, or water-soluble fractions. Mortality in the acetone-Triton X–100-water-treated controls for all species in this study was less than 2%.

**Table 1:** Toxicity of each fraction obtained from the solvent partitioning of the methanol extract of the *Magnolia denudata* seeds to third-instar larvae of two mosquito species during a 24 h exposure.

Material	*Culex pipiens pallens* larvae		*Aedes aegypti* larvae	
	Slope ± SE	LC_50_, mg/L (95% CI)	Slope ± SE	LC_50_, mg/L (95% CI)
Methanol extract	4.5 ± 0.63	43.30 (29.44–66.60)	2.7 ± 0.33	58.70 (56.29–61.30)
Hexane-soluble fraction	3.3 ± 0.63	21.21 (16.52–26.26)	3.1 ± 0.41	26.10 (19.57–33.95)
Chloroform-soluble fraction		>100		>100
Ethyl acetate-soluble fraction		>100		>100
Butanol-soluble fraction		>100		>100
Water-soluble fraction		>100		>100

**Figure 1:**
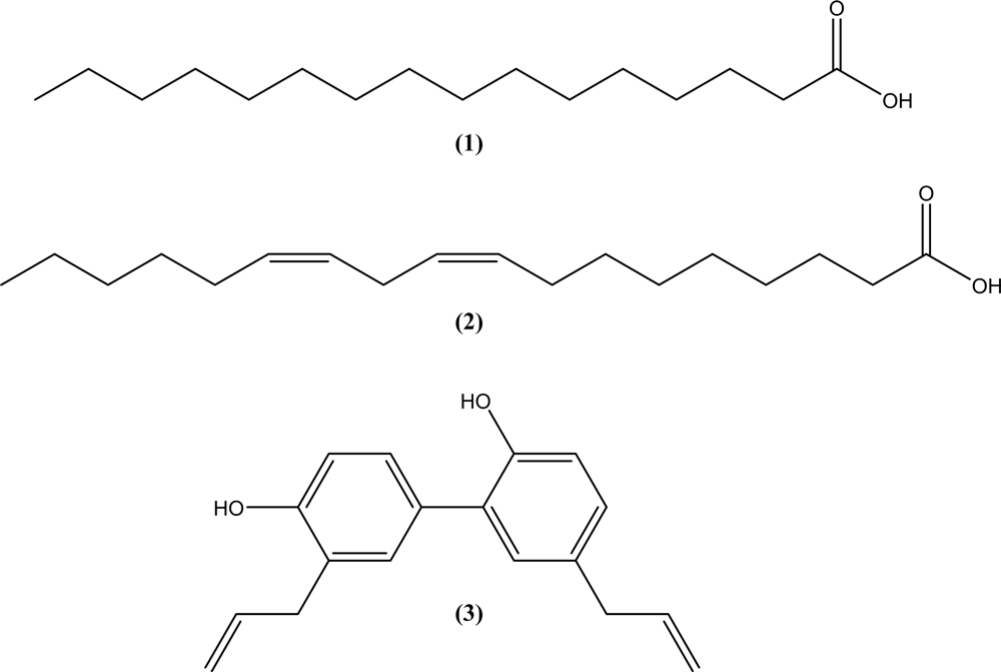
Structures of palmitic acid, linoleic acid, and honokiol. These compounds were identified in the seeds of *Magnolia denudata* in this study. The chemical formula of the saturated fatty acid palmitic acid (**1**) is C_16_H_32_O_2_, with a molar mass of 256.43 g/mol. The chemical formula of the unsaturated fatty acid linoleic acid (**2**) is C_18_H_32_O_2_, with a molar mass of 280.45 g/mol. The chemical formula of the neolignan honokiol (**3**) is C_18_H_18_O_2_, with a molar mass of 266.33 g/mol.

Contact mortality bioassay-guided fractionation of the *M*. *denudata* seeds led to the identification of three active compounds through spectroscopic analyses, including electron ionized mass spectrometry (EI-MS) and nuclear magnetic resonance (NMR) spectroscopy. The three larvicidal constituents were palmitic acid (hexadecanoic acid) (**1**), linoleic acid ((9*Z*,12*Z*)-9,12-octadecadienoic acid) (**2**), and honokiol (2-(4-hydroxy-3-prop-2-enyl-phenyl)-4-prop-2-enyl-phenol) (**3**) (Fig. 1). Palmitic acid (**1**) was identified based on the following characteristics: a white crystal; ultraviolet (UV) (ethanol): λ_max_ nm = 254; EI-MS (70 eV), *m*/*z* (% relative intensity): 256 [M]^+^ (100), 213 (49), 185 (29), 171 (26), 157 (26),143 (12), 129 (65), 115 (23), 97 (33), 83 (36), 73 (91), 60 (69) (see Supplementary Fig. S1); ^1^H NMR (CDCl_3_, 600 MHz): δ 0.89 (3 H, t), 1.30 (17 H, m), 1.30 (2 H, m), 1.31 (2 H, m), 1.31 (2 H, m), 1.33 (2 H, m), 1.60 (2 H, m), 2.27 (2 H, t) (see Supplementary Fig. S2); and ^13^C NMR (CDCl_3_, 150 MHz): δ 14.5 (C-16), 23.8 (C-15), 26.7 (C-5), 27.3 (C-3, C-4), 28.3 (C-9, C-10, C-11), 30.1 (C-8), 30.4 (C-13), 30.6 (C-12), 30.9 (C-6, C-7), 35.1 (C-14), 35.2 (C-2), 177.9 (C-1) (see Supplementary Fig. S3). Linoleic acid (**2**) was characterized based on the following: a colorless oil; UV (methanol): λ_max_ nm = 290; EI-MS (70 eV), *m*/*z* (% relative intensity): 280 [M]^+^ (65), 137 (15), 123 (22), 110 (32), 108 (11), 96 (54), 95 (70), 82 (73), 81 (100), 67 (87), 55 (54) (see Supplementary Fig. S4); ^1^H NMR (CDCl_3_, 600 MHz): δ 0.87 (3 H, s), 1.31 (14 H, m), 1.62 (2 H, m), 2.05 (2 H, d, *J* = 6.6 Hz), 2.05 (2 H, d, *J* = 13.3 Hz), 2.34 (2 H, t, *J* = 7.5 Hz), 2.78 (2 H, t, *J* = 5.9 Hz), 3.67 (1 H, s), 5.31 (2 H, m), 5.35 (2 H, m), (see Supplementary Fig. S5); ^13^C NMR (CDCl_3_, 150 MHz): δ 14.1 (C-18), 22.6 (C-17), 24.6 (C-3), 25.6 (C-11), 27.2 (C-8), 27.3 (C-14), 27.8 (C-12), 29.0 (C-4), 29.1 (C-5), 29.2 (C-15), 29.3 (C-6), 29.6 (C-7), 30.2 (C-13), 31.5 (C-16), 34.0 (C-2), 128.0 (C-10), 130.0 (C-9), 180.1 (C-1) (see Supplementary Fig. S6); and distortion-less enhancement by polarization transfer (DEPT) spectra (see Supplementary Fig. S7). Honokiol (**3**) was characterized based on the following: a colorless solid; UV (methanol): λ_max_ nm = 293); EI-MS (70 eV), *m*/*z* (% relative intensity): 266 [M]^+^ (100), 237(18), 224 (8), 197 (8), 184 (8), 152 (3), 133 (4), 105 (2), 77 (1) (see Supplementary Fig. S8); ^1^H NMR (CDCl_3_, 600 MHz): δ 3.30 (2 H, d, *J* = 6 Hz, H-7), 3.37 (2 H, d, *J* = 6.6 Hz, H-7′), 5.07 (2 H, m), 5.92 (4 H, m, H-9, H-9′), 6.78 (2 H, t, H-8, H-8′), 6.90 (1 H, d, *J* = 2.1 Hz, H-3), 6.90 (1 H, dd, *J* = 2.1 and 2.2 Hz, H-3′), 6.97 (1 H, d, *J* = 2.2 Hz, H-6′), 7.19 (1 H, d, *J* = 2.2 Hz, H-4), 7.20 (1 H, d, *J* = 2.6 Hz, H-6), 7.22 (1 H, d, *J* = 2.1 Hz, H-2′) (see Supplementary Fig. S9); ^13^C NMR (CDCl_3_, 150 MHz): δ 35.5 (C-7), 40.6 (C-7′), 115.5 (C-6), 115.5 (C-2′), 115.6 (C-9), 115.6 (C-9′), 117.0 (C-8), 127.4 (C-4), 128.8 (C-4′), 129.3 (C-1′), 129.3 (C-5), 130.1 (C-6′), 132.2 (C-2), 132.6 (C-8), 138.7 (C-3), 139.7 (C-3′), 153.5 (C-5), 155.3 (C-1) (see Supplementary Fig. S10); and DEPT spectra (see Supplementary Fig. S11).

### Larvicidal activity of the isolated compounds

The toxicity of the three isolated compounds (palmitic acid, linoleic acid, and honokiol), their corresponding pure organic compounds, and the OP temephos, which was used as a positive control, against third-instar larvae of the susceptible KS-CP strain of *Cx*. *pipiens pallens* was evaluated (Table 2). The responses varied according to the compounds that were examined. As judged by the 24 h LC_50_ values, natural and pure organic honokiol had similar toxicities against KS-CP larvae, indicating that the activity of the methanol-extracted honokiol was purely due to honokiol. Similar results were obtained from natural and pure organic linoleic acid and palmitic acid. Natural honokiol (LC_50_, 6.32 mg/L) and linoleic acid (6.97 mg/L) were the most toxic constituents and were 390 and 430 times less toxic than temephos, respectively. The LC_50_ of palmitic acid was 32.56 mg/L.

**Table 2:** Toxicity of the *Magnolia denudata* seed constituents, their pure organic compounds, and larvicide temephos to third-instar larvae of insecticide-susceptible *Culex pipiens pallens* during a 24 h exposure.

Compound	Slope ± SE	LC_50_ (mg/L) (95% CI)	LC_90_ (mg/L) (95% CI)	χ^2^	*P*-value
Natural honokiol	2.4 ± 0.30	6.32 (5.07–7.60)	18.78 (15.30–25.99)	3.10	0.978
Pure honokiol	2.1 ± 0.30	6.38 (5.01–7.78)	19.30 (15.64–27.01)	2.48	0.991
Natural linoleic acid	4.4 ± 0.59	6.97 (6.21–7.68)	19.41 (15.94–26.34)	4.39	0.927
Pure linoleic acid	2.5 ± 0.22	8.36 (6.09–10.40)	47.79 (33.03–46.79)	4.38	0.986
Natural palmitic acid	3.1 ± 0.38	32.56 (28.22–37.19)	112.3 (88.96–156.2)	4.76	0.923
Pure palmitic acid	2.4 ± 0.25	34.92 (29.48–40.76)	119.3 (94.38–166.4)	4.21	0.988
Temephos	1.9 ± 0.21	0.0162 (0.0132–0.0196)	0.0749 (0.0551–0.1177)	3.93	0.991

Against third-instar larvae of insecticide-susceptible *Ae*. *aegypti* (Table 3), natural and pure organic honokiol did not differ significantly in their respective toxicities from each other. Similar results were also observed with natural and pure organic linoleic acid and palmitic acid. Natural honokiol (24 h LC_50_, 6.51 mg/L) and linoleic acid (7.19 mg/L) were the most toxic constituents and were 521 and 575 times less toxic than temephos, respectively. The toxicity of palmitic acid was the lowest of any of the constituents tested.

**Table 3:** Toxicity of the *Magnolia denudata* seed constituents, their pure organic compounds, and larvicide temephos to third-instar larvae of insecticide-susceptible *Aedes aegypti* during a 24 h exposure.

Compound	Slope ± SE	LC_50_ (mg/L) (95% CI)	LC_90_ (mg/L) (95% CI)	χ^2^	*P*-value
Natural honokiol	2.3 ± 0.37	6.51 (5.19–7.89)	22.59 (17.70–34.15)	1.47	0.999
Pure honokiol	2.2 ± 0.36	6.48 (5.11–7.90)	23.84 (18.41–37.36)	1.09	0.999
Natural linoleic acid	4.4 ± 0.59	7.19 (6.38–7.95)	27.04 (20.52–43.96)	1.00	0.999
Pure linoleic acid	2.1 ± 0.29	7.34 (5.58–8.92)	29.70 (23.19–43.79)	4.11	0.917
Natural palmitic acid	3.1 ± 0.38	33.54 (29.06–38.38)	129.3 (99.59–189.2)	3.93	0.991
Pure palmitic acid	2.3 ± 0.25	36.49 (29.30–44.83)	204.1 (139.4–378.1)	2.17	0.999
Temephos	1.7 ± 0.21	0.0125 (0.0098–0.0154)	0.0658 (0.0479–0.1059)	6.87	0.908

The toxic effects of all of the compounds on third-instar larvae of insecticide-susceptible *Ae*. *albopictus* were also compared (Table 4). Natural honokiol (24 LC_50_, 6.13 mg/L) and linoleic acid (7.28 mg/L) were 464 and 552 times less toxic than temephos against *Ae*. *albopictus* larvae, respectively. Palmitic acid was the least toxic constituent. Interestingly, the toxicity of all compounds was virtually identical against *Ae*. *albopictus* and *An*. *sinensis* larvae (Table 5), indicating a lack of cross-resistance in the larval *An*. *sinensis*^31^.

**Table 4:** Toxicity of the *Magnolia denudata* seed constituents and larvicide temephos to third-instar larvae of insecticide-susceptible *Aedes albopictus* during a 24 h exposure.

Compound	Slope ± SE	LC_50_ (mg/L) (95% CI)	LC_90_ (mg/L) (95% CI)	χ^2^	*P*-value
Honokiol	2.9 ± 0.52	6.13 (5.19–7.38)	16.52 (13.74–21.90)	4.07	0.943
Linoleic acid	4.4 ± 0.59	7.28 (6.53–8.00)	27.81 (20.95–46.02)	1.22	0.999
Palmitic acid	3.1 ± 0.38	36.91 (31.30–41.64)	135.1 (104.3–196.9)	4.28	0.987
Temephos	1.6 ± 0.21	0.0132 (0.0103–0.0164)	0.0778 (0.0547–0.1343)	4.68	0.912

**Table 5:** Toxicity of the *Magnolia denudata* seed constituents and larvicide temephos to third-instar larvae of insecticide-resistant *Anopheles sinensis* during a 24 h exposure.

Compound	Slope ± SE	LC_50_ (mg/L) (95% CI)	LC_90_ (mg/L) (95% CI	χ^2^	*P*-value
Honokiol	2.0 ± 0.35	7.37 (5.45–9.04)	31.45 (22.90–56.82)	1.03	0.999
Linoleic acid	3.9 ± 0.56	7.50 (6.80–8.29)	16.04 (14.05–19.06)	2.30	0.986
Palmitic acid	2.1 ± 0.22	47.58 (40.24–55.68)	192.8 (148.1–282.2)	2.26	0.999
Temephos	2.2 ± 0.15	0.40 (0.34–0.47)	1.39 (1.05–2.13)	3.04	0.997

### Structure–activity relationship

**Figure 2:**
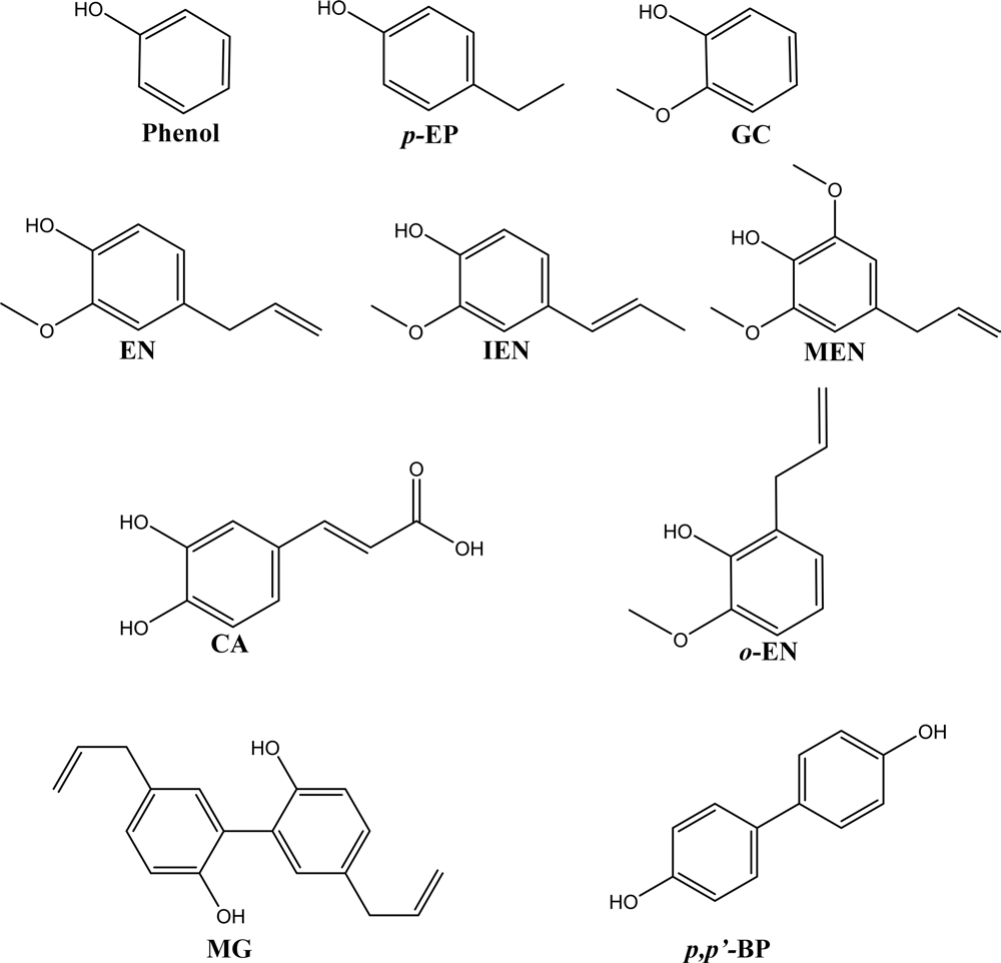
Structures of 10 structurally related compounds of honokiol. Phenol, *p*-ethylphenol (*p*-EP), guaiacol (GC), eugenol (EN), isoeugenol (IEN), caffeic acid (CA), *o*-eugenol (*o*-EN), magnolol (MG), methoxyeugenol (MEN), and *p*,*p′*-biphenol (*p*,*p′*-BP).

Because of the potent larvicidal activity of honokiol, comparisons were made to determine the toxicity differences due to the chemical structures and functional groups of its 10 analogs (Fig. 2) using their respective toxicity data (Table 6). Against third-instar *Cx*. *pipiens pallens* larvae, phenol, with a basic structure, exhibited moderate toxicity (24 h LC_50_, 41.67 mg/L). Honokiol was the most toxic compound, followed by *o*-eugenol, *p*,*p*′-biphenol, *p*-ethylphenol, and methoxyeugenol (LC_50_, 6.32–8.40 mg/L), and these compounds did not significantly differ in toxicity from each other. Magnolol (LC_50_, 26.00 mg/L) had significantly more pronounced toxicity than phenol. The LC_50_ of eugenol and isoeugenol was 74.35 and 79.72 mg/L, respectively. The toxicities of guaiacol and caffeic acid were the lowest of all of the constituents examined. Similar differences in the responses of third-instar *Cx*. *pipiens pallens* larvae to the 11 compounds were also observed in third-instar *Ae*. *aegypti* larvae (Table 7).

**Table 6:** Toxicity of honokiol, its 10 structurally related compounds, and organophosphorus insecticide dichlorvos to third-instar *Culex pipiens pallens* larvae during a 24 h exposure.

Compound	Slope ± SE	LC_50_ (mg/L) (95% CI)	LC_90_ (mg/L) (95% CI)	χ^2^	*P*-value
Honokiol	2.4 ± 0.30	6.32 (5.07–7.60)	18.78 (15.30–25.99)	3.10	0.978
*o*-Eugenol	2.6 ± 0.38	7.46 (5.98–8.78)	23.13 (18.42–33.49)	2.94	0.982
*p*,*p*′-Biphenol	2.5 ± 0.37	7.52 (5.99–8.88)	23.97 (18.97–35.16)	1.00	0.999
*p*-Ethylphenol	2.5 ± 0.37	8.30 (6.80–9.72)	25.86 (20.36–38.21)	3.78	0.956
Methoxyeugenol	2.6 ± 0.37	8.40 (6.91–9.80)	25.52 (20.27–36.92)	1.06	0.999
Magnolol	2.6 ± 0.35	26.00 (22.17–30.75)	80.42 (63.28–117.4)	2.34	0.993
Phenol	1.9 ± 0.24	41.67 (34.56–50.07)	185.1 (133.5–306.6)	1.84	0.999
Eugenol	2.9 ± 0.39	74.35 (64.14–85.27)	143.3 (127.2–170.3)	5.40	0.965
Isoeugenol	2.7 ± 0.39	79.72 (68.33–92.65)	138.1 (124.4–164.5)	3.67	0.960
Guaiacol	4.5 ± 0.55	107.8 (99.62–117.6)	205.6 (176.3–260.9)	3.08	0.997
Caffeic acid	4.1 ± 0.56	119.3 (108.5–131.9)	245.6 (208.1–323.3)	2.16	0.999
Dichlorvos	2.0 ± 0.22	0.103 (0.077–0.128)	0.504 (0.380–0.761)	3.84	0.993

**Table 7:** Toxicity of honokiol, its 10 structurally related compounds, and organophosphorus insecticide dichlorvos to third-instar *Aedes aegypti* larvae during a 24 h exposure.

Compound	Slope ± SE	LC_50_ (mg/L) (95% CI)	LC_90_ (mg/L) (95% CI)	χ^2^	*P*-value
Honokiol	2.3 ± 0.30	6.51 (5.19–7.89)	20.91 (16.72–30.14)	1.81	0.997
*o*-Eugenol	2.9 ± 0.38	8.84 (7.48–10.16)	24.01 (19.52–33.06)	2.01	0.995
*p,p’*-Biphenol	2.4 ± 0.36	8.34 (6.74–9.84)	27.76 (21.50–42.52)	1.77	0.997
*p*-Ethylphenol	2.4 ± 0.36	9.03 (7.38–10.64)	30.57 (23.29–48.51)	2.53	0.990
Methoxyeugenol	2.5 ± 0.36	9.15 (7.55–10.72)	29.46 (22.83–44.88)	1.68	0.998
Magnolol	4.5 ± 0.53	25.70 (21.00–30.50)	83.14 (64.68–124.4)	3.00	0.981
Phenol	1.8 ± 0.23	43.22 (35.62–52.46)	207.1 (145.1–363.6)	1.12	0.999
Eugenol	4.7 ± 0.52	75.76 (68.75–82.02)	141.4 (126.3–166.2)	4.60	0.982
Isoeugenol	5.6 ± 0.87	81.26 (71.39–88.47)	137.2 (124.3–161.2)	3.11	0.978
Guaiacol	4.4 ± 0.56	115.4 (106.3–127.2)	223.1 (188.3–292.1)	5.95	0.948
Caffeic acid	4.6 ± 0.57	124.5 (115.2–134.8)	235.9 (204.2–296.1)	1.58	0.999
Dichlorvos	2.0 ± 0.22	0.158 (0.128–0.190)	0.660 (0.505–0.963)	3.84	0.993

Multiple regression analysis of the contact toxicities of compounds against larvae of the two mosquito species was performed using their LC_50_ values and the values of the physical parameters (molecular weight (MW), log *P*, and molecular refraction (MR)) of the 11 compounds (R^2^ = 0.407 for larval *Cx*. *pipiens pallens*; R^2^ = 0.418 for larval *Ae*. *aegypti*). Correlation coefficient (*r*) analysis showed that the log *P* was negatively correlated with the LC_50_ for larval *Cx*. *pipiens pallens* (*r* = −0.584 (*P* = 0.059)) and the MW and MR were only loosely negatively correlated with the LC_50_ (MW, *r* = −0.281 (*P* = 0.403); MR, *r* = −0.364 (*P* = 0.271)). Similar results were also observed in larval *Ae*. *aegypti* (log *P*, *r* = −0.595 (*P* = 0.054); MW, *r* = −0.291 (*P* = 0.386); and MR, *r* = −0.375 (*P* = 0.256)). There were no differences in the correlation coefficients between larval *Cx*. *pipiens pallens* and *Ae*. *aegypti*.

### Acetylcholinesterase inhibition

**Figure 3:**
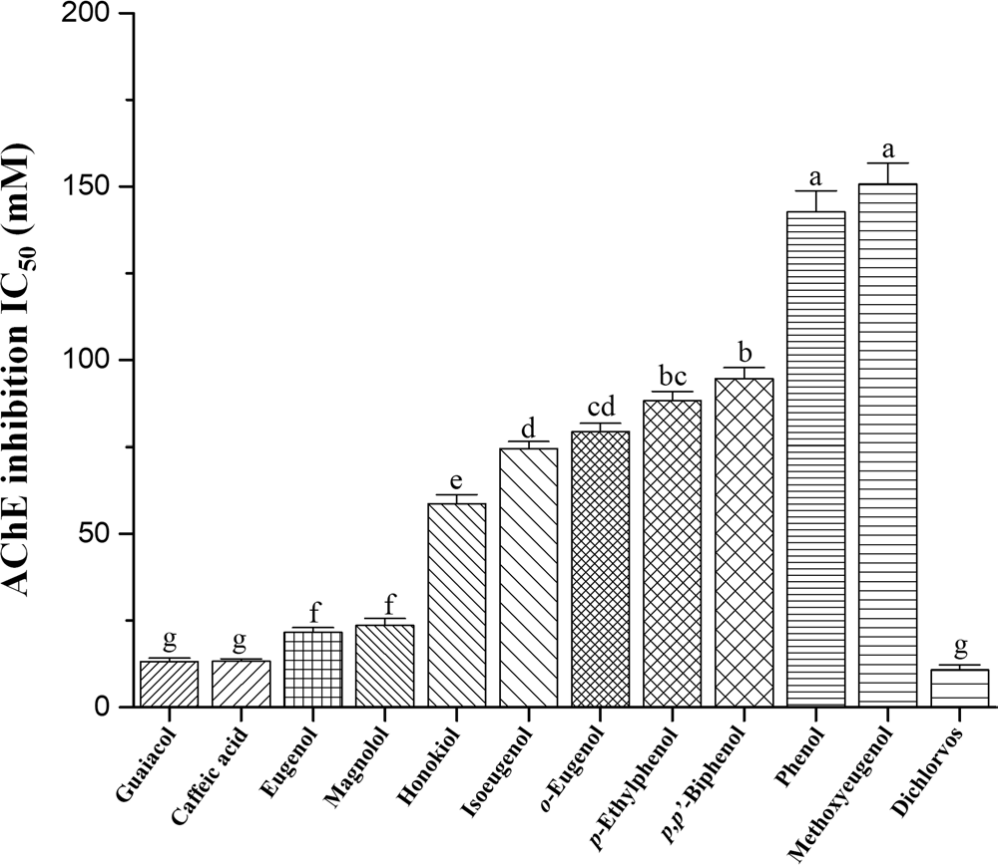
Inhibitory effects on acetylcholinesterase. Inhibition of acetylcholinesterase (AChE) extracted from third-instar *Aedes aegypti* larvae by isolated neolignan honokiol, magnolol, a structural isomer of honokiol, and nine structurally related phenol compounds of honokiol was determined by acetylthiocholine iodide hydrolysis at 30 °C and pH 8.0 as described in the Methods section. Each bar represents the mean ± standard error of triplicate samples from three independent experiments (*P* = 0.05, using Bonferroni’s multiple comparisons test).

The *in vitro* acetylcholinesterase (AChE) inhibitory activity of honokiol, its 10 structurally related compounds, and the OP dichlorvos, which was used as a positive control, was evaluated using AChE from third-instar *Ae*. *aegypti* larvae (Fig. 3). The responses varied according to the compounds that were examined. Based on the IC_50_ values, there were significant differences (*F* = 866.67; df = 11, 24; *P* < 0.0001) in inhibition of AChE by the test compounds. Guaiacol and caffeic acid were the most potent AChE inhibitors (IC_50_, 13 mM), and the inhibitory activity of these compounds and dichlorvos did not differ significantly. Eugenol and magnolol (IC_50_, 22 and 24 mM) had significantly more pronounced inhibition of AChE than honokiol (59 mM). The IC_50_ of isoeugenol, *o*-eugenol, *p*-ethylphenol, and *p,p′*-biphenol was between 75 and 95 mM. The AChE inhibitory activities of methoxyeugenol and phenol were the lowest of all of the compounds that were examined.

### Effects on cyclic AMP production

**Figure 4:**
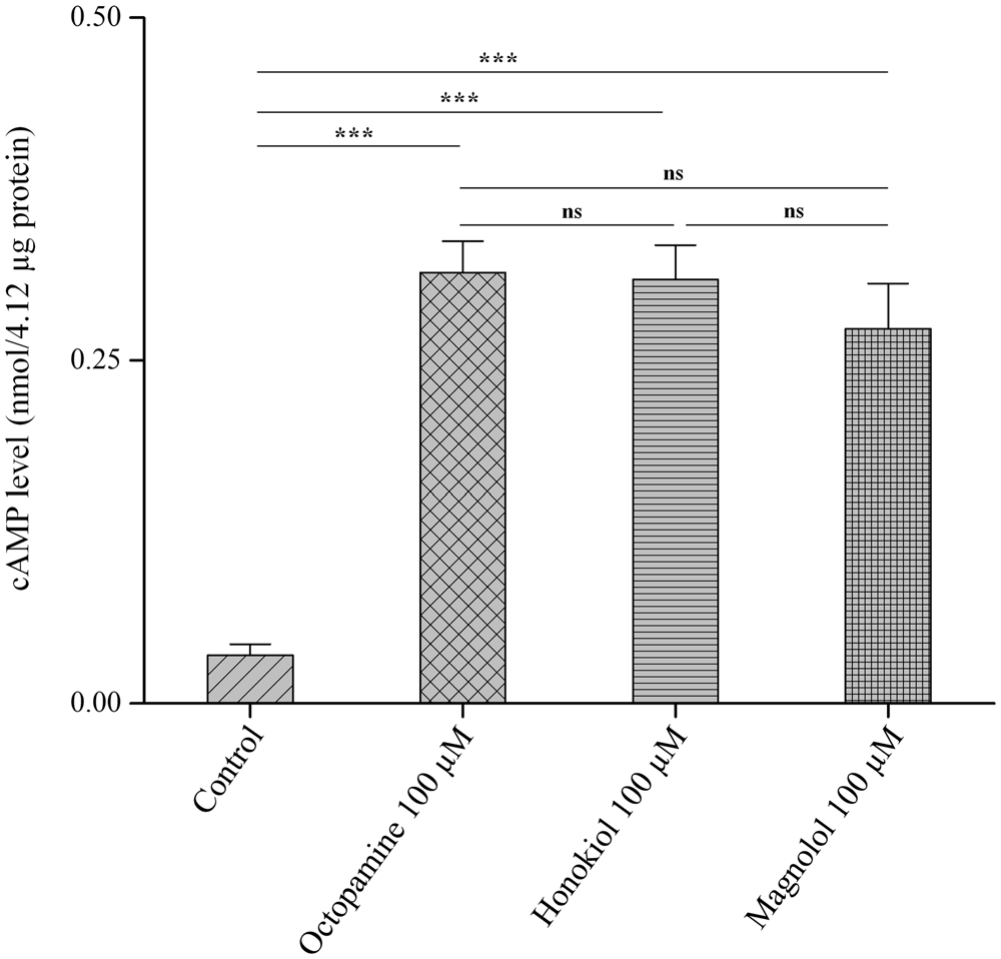
Effects on cyclic AMP levels. A whole-body homogenate from third-instar *Aedes aegypti* larvae was tested for adenylate cyclase activity, as described in the Methods section, in the presence of honokiol (100 µM) and magnolol (100 µM). The effects of the neolignans on cyclic AMP (cAMP) levels of the homogenate were compared with those of octopamine (100 µM) alone. Data were expressed as nmol/4.12 μg protein. Each bar represents the mean ± standard error of duplicate samples from three independent experiments (***P < 0.001; ns, no significant difference, using Bonferroni’s multiple comparisons test).

The effects of the two neolignans on the cyclic AMP (cAMP) levels of whole-body homogenates from third-instar *Ae*. *aegypti* larvae were elucidated and compared with those induced by octopamine alone (Fig. 4). As judged by the preliminary test results, the cAMP levels induced by honokiol and magnolol were determined to be 100 μM, because no significant differences in the levels were observed among the three concentrations examined (50, 100, and 200 μM). There were significant differences (*F* = 869.51; df = 3, 8; *P* < 0.0001) in the cAMP levels for the different test compounds. At a concentration of 100 μM, the cAMP levels induced by honokiol and magnolol were significantly higher than that induced by the control group. The cAMP levels induced by the two neolignans and octopamine did not differ significantly.

### Pathological symptoms associated with the two neolignans

**Figure 5:**
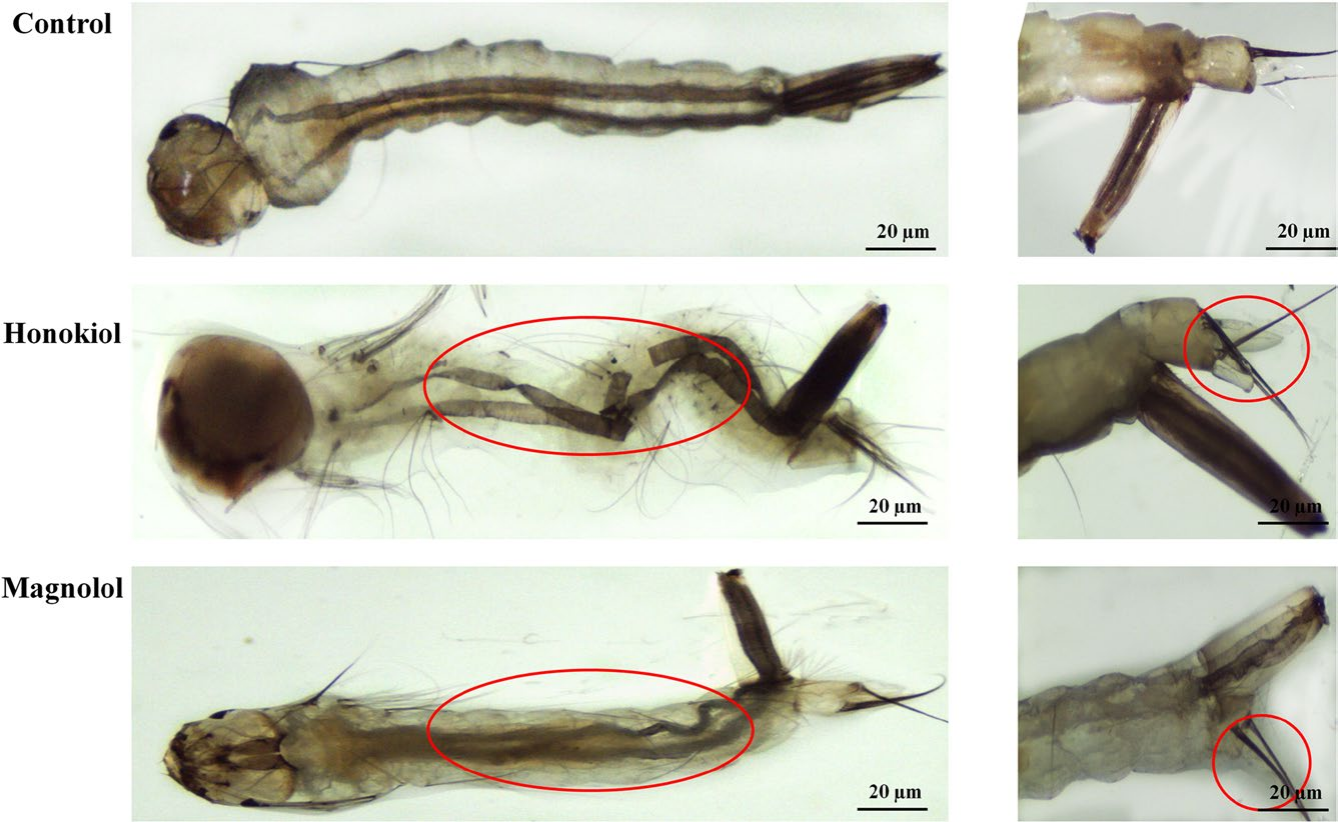
Light micrographs of midgut, thorax, and anal gill parts of larval *Aedes aegypti*. Third-instar *Ae*. *aegypti* larvae were placed into paper cups containing a methanol–Triton X-100 solution in distilled water with an LC_50_ of honokiol (6.5 mg/L) or magnolol (25 mg/L) for 24 h. The morphology of the whole body of the larvae was observed with a stereo microscope (35× magnification). (**A**) Untreated control larvae showed normal appearances of typical structures, with well-developed, distinguished head, thorax, and abdominal regions. Honokiol-treated larvae were completely damaged with indistinct appearances, particularly in the thorax and abdominal region segments. In particular, the midgut region was completely ruptured, and the midgut contents oozed out from the body. Magnolol-treated larvae showed shrunken bodies with no distinguishable abdominal segments. Intrinsic body fluid contents became dark, and there were unusually elongated digestive tracts with damaged interior tissues. (**B**) Control larvae showed well-developed anal gills and anal gill cells. In larvae treated with honokiol, the anal gill from one side was swollen and reduced in length and the other one was completely damaged. In larvae treated with magnolol, the anal gill region was completely damaged, with outer cuticle membranes with indistinct anal gills and no internal body fluid. All experiments were performed in duplicate, and 20 mosquito larvae were used in each replicate. More than 10 live larvae from control and treated groups were randomly collected and used for analysis.

The normal morphology of the entire body of the untreated (control) third-instar *Ae*. *aegypti* larvae showed a common appearance of the typical structures, with well-developed, distinct heads, thoracic regions, and abdominal regions (Fig. 5A). However, the whole body of the larval *Ae*. *aegypti* treated with honokiol (6.5 mg/L) was severely damaged, with an indistinct appearance of the thorax, midgut, and abdominal regions. The honokiol-treated larvae exhibited dark spots over the entire body. In particular, the midgut region was completely ruptured, and the midgut contents oozed out from the body. Likewise, larval *Ae*. *aegypti* treated with magnolol (25 mg/L) showed a shrunken body with no distinct abdominal segments (from the 3rd to 7th segments). The intrinsic body fluid contents became dark, and there was an unusual elongated digestive tract with damaged interior tissues. The anal gill region of control *Ae*. *aegypti* larvae showed well-developed anal gills with thick cuticle-covered undamaged anal gill cells (Fig. 5B). In larvae treated with honokiol, the anal gill from one side was swollen and reduced from its normal length, and the other one was completely damaged. The anal gill region of larvae treated with magnolol showed completely damaged outer cuticle membranes with undistinguishable anal gills with no internal body fluid.

### Histopathological effects of two neolignans visualized by Carson’s staining

**Figure 6:**
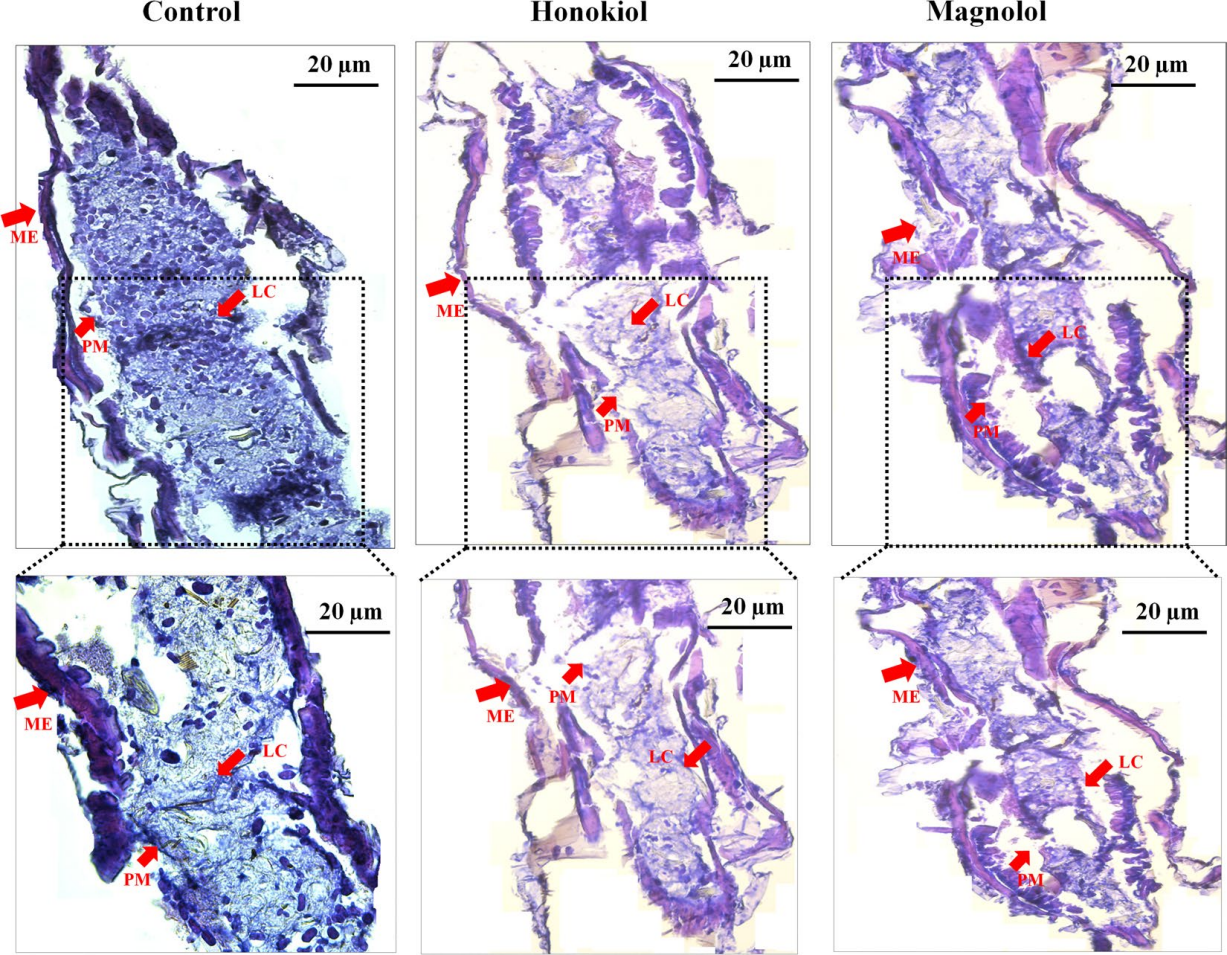
Histology of midgut regions of larval *Aedes aegypti*. Third-instar *Ae*. *aegypti* larvae were placed into paper cups containing a methanol–Triton X-100 solution in distilled water with an LC_50_ of honokiol (6.5 mg/L) or magnolol (25 mg/L) for 24 h. Carson’s trichrome staining was performed as described in the Methods section. Observations were taken of 15 larvae under the microscope. (**A**) Control larvae. The well-developed midgut regions of control larvae showed distinct midgut epithelium (ME) and peritrophic membranes (PMs), which enclosed the rich lumen contents (LCs) in the midgut region. (**B**) Honokiol-treated larvae. The midgut regions of the larvae showed a damaged midgut epithelial layer with smashed PMs consisting of lumen debris. (**C**) Magnolol-treated larvae. The midgut regions of the larvae showed indistinguishable midgut epithelial layers as well as PMs with damaged LC.

Histological observation of the whole bodies of third-instar *Ae*. *aegypti* larvae in the control group showed a typical appearance of the thoracic and midgut regions. The well-developed midgut regions of untreated larvae showed distinct, single-layered midgut epithelium (ME) and peritrophic membranes (PMs) and had rich lumen contents (LCs) in the midgut region (Fig. 6A). By contrast, the midgut region of *Ae*. *aegypti* larvae treated with honokiol (6.5 mg/L) showed indistinct damaged midgut epithelial layers. The PM next to the epithelial layer, which surrounded the LCs, was collapsed; this led to luminal debris and indistinct nuclei of epithelial cells in the midgut region (Fig. 6B). Similarly, *Ae*. *aegypti* larvae treated with magnolol (25 mg/L) also showed indistinct midgut epithelial layers and PMs. Both midgut epithelial layers and PM cells were indistinct, with damaged LCs. Debris of the epithelial and midgut contents without proper nuclei were observed (Fig. 6C).

### Histopathological effects of two neolignans on midguts as determined via transmission electron microscopy

**Figure 7:**
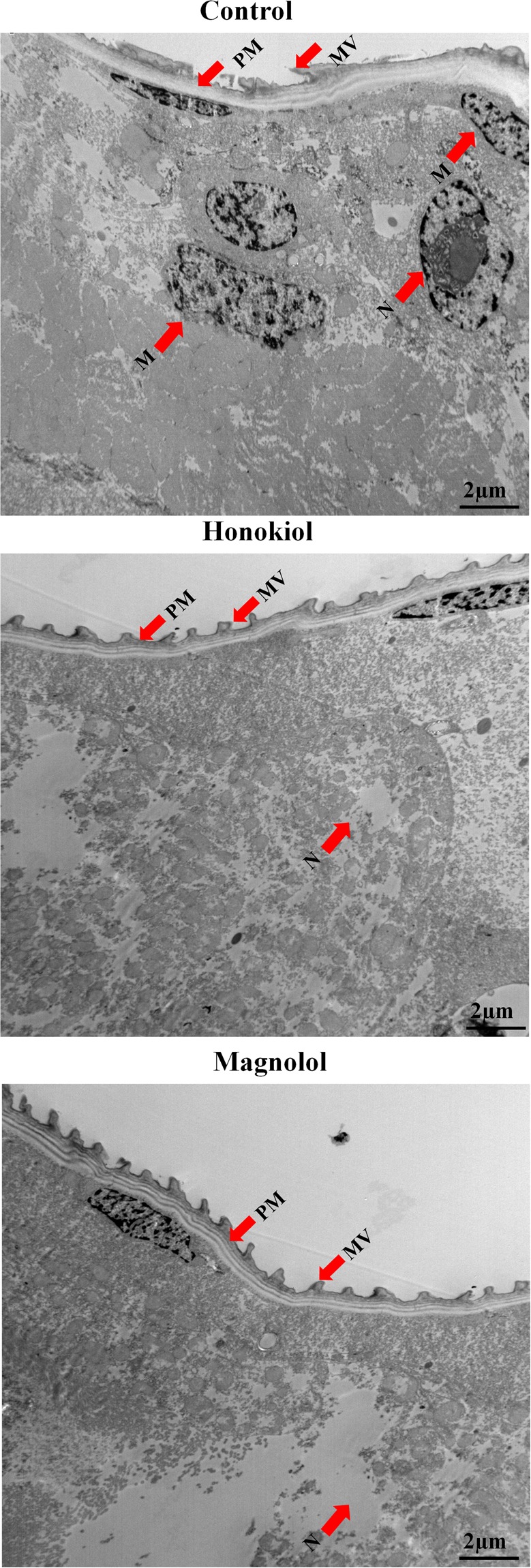
Transmission electronic micrographs of midgut regions of larval *Aedes aegypti*. Third-instar *Ae*. *aegypti* larvae were placed into paper cups containing a methanol–Triton X-100 solution in distilled water with an LC_50_ of honokiol (6.5 mg/L) or magnolol (25 mg/L) for 24 h. (**A**) Control larvae. A transmission electron micrograph revealed that the peritrophic membrane (PM) in the midgut regions of the larvae enclosed the midgut lumen contents (LCs) and consisted of numerous microvilli (MV) from the outside. The LC consists of the cytoplasm and numerous cell organelles surrounded by a plasma membrane. There was no damage to cell organelles in the cytoplasm, including prominent large central nuclei (approximately 15 µm diameter), mitochondria, and other cell organelles. (**B**) Honokiol-treated larvae. The neolignan destroyed all cellular material and extruded masses in the cytoplasm. In particular, mitochondria and large prominent nuclei were indistinguishable from damaged cellular contents. There was no distinct nuclear material inside the nucleus. (**C**) Magnolol-treated larvae. These showed complete disappearance of the nucleus, as well as cytoplasmic contents. Nuclei and mitochondria were completely absent. Other cytoplasmic organelles were damaged with indistinct appearances.

The midgut regions of third-instar *Ae*. *aegypti* larvae untreated and treated with honokiol (6.5 mg/L) or magnolol (25 mg/L) were observed via TEM. A PM in the midgut regions of control *Ae*. *aegypti* larvae enclosed the midgut LCs and consisted of numerous microvilli (MV) from the outside. The midgut regions of control larvae were composed of a well-spaced cytoplasm and numerous cell organelles that were surrounded by a plasma membrane. There was no damage to the cell organelles in the cytoplasm, and there were large, prominent central nuclei (approximately 15 µm diameter), mitochondria, and other cell organelles (Fig. 7A). However, honokiol-treated larvae showed devastated cellular material and extruded masses in the cytoplasm. In particular, mitochondria and large prominent nuclei were undistinguishable from damaged cellular contents. There was no distinct nuclear material inside the nucleus. Some midgut LCs were extruded from the cytoplasm (Fig. 7B). However, magnolol-treated larvae showed a complete disappearance of nuclei as well as cytoplasmic contents. The cellular organelles, such as the nucleus and mitochondria, completely disappeared. In addition, other cytoplasmic organelles were also severely damaged with indistinguishable appearances (Fig. 7C).

### Histopathological effects of two neolignans on anal gills as determined via transmission electron microscopy

**Figure 8:**
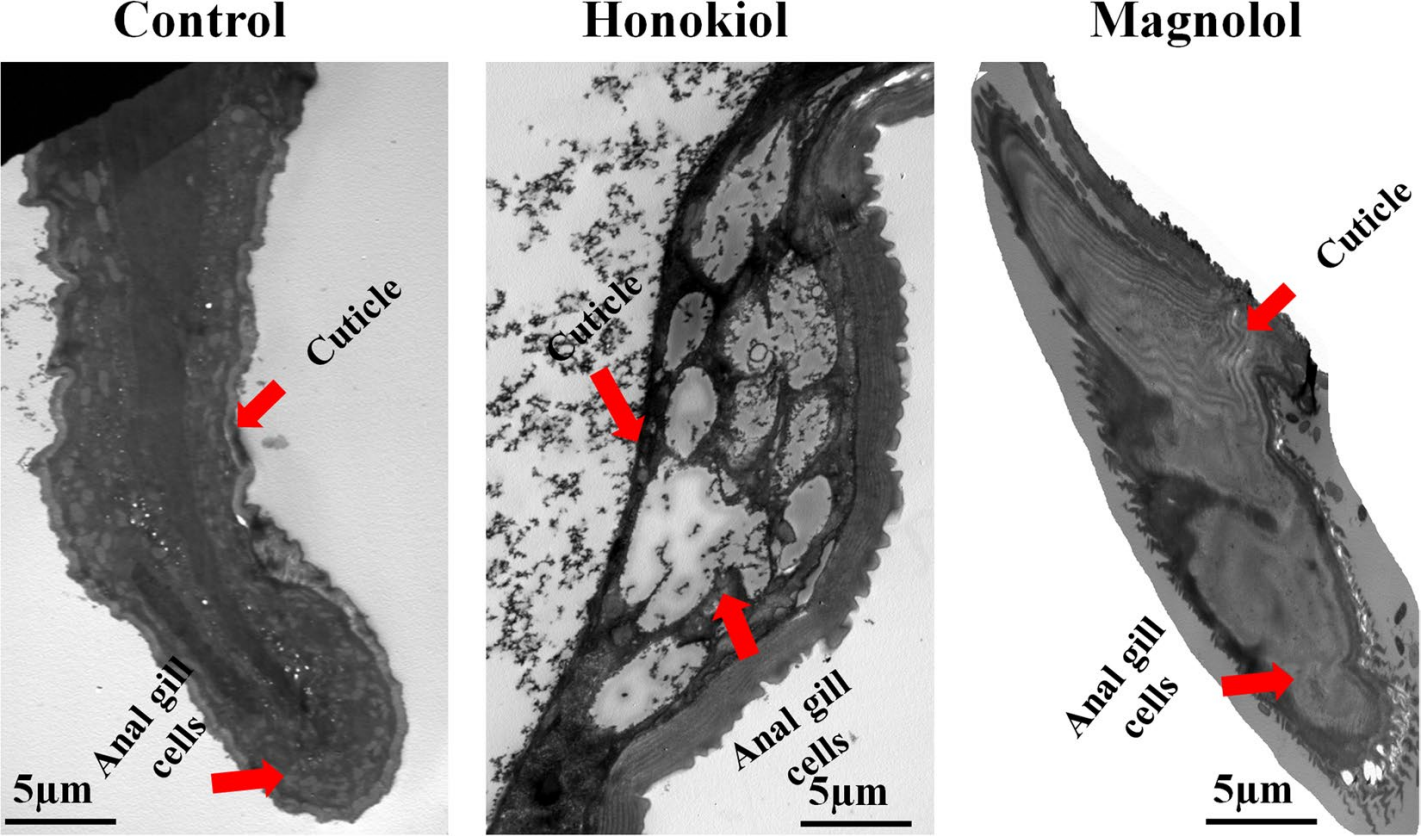
Transmission electronic micrographs of the anal gill regions of larval *Aedes aegypti*. Third-instar *Ae*. *aegypti* larvae were placed into paper cups containing a methanol–Triton X-100 solution in distilled water with an LC_50_ of honokiol (6.5 mg/L) or magnolol (25 mg/L) for 24 h. (**A**) Control larvae. Transmission electronic micrography (TEM) analysis revealed that the anal gills of the control larva were surrounded by thick cuticles and that the inner surfaces of the cytoplasm became fuller, with anal gill cells and fluid-filled tracheoles. (**B**) Honokiol-treated larvae. Histopathological observation of the larvae indicated cytoplasmic disruption in anal gills with damaged anal gill cells. (**C**) Magnolol-treated larvae. TEM showed damaged outer membranes of thick cuticles, which led to internal cytoplasmic destruction.

TEM revealed that the anal gills of control third-instar *Ae*. *aegypti* larvae were surrounded by thick cuticles and that the inner surface of the cytoplasm became more consistent with fluid-filled tracheoles (Fig. 8A). Histopathological observation of honokiol-treated larvae indicated cytoplasmic disruption with damaged anal gill cells (Fig. 8B). However, magnolol-treated larvae showed damaged outer membranes of thick cuticles, which led to internal cytoplasmic destruction (Fig. 8C).

### Effects of two neolignans on target gene expression levels

**Figure 9:**
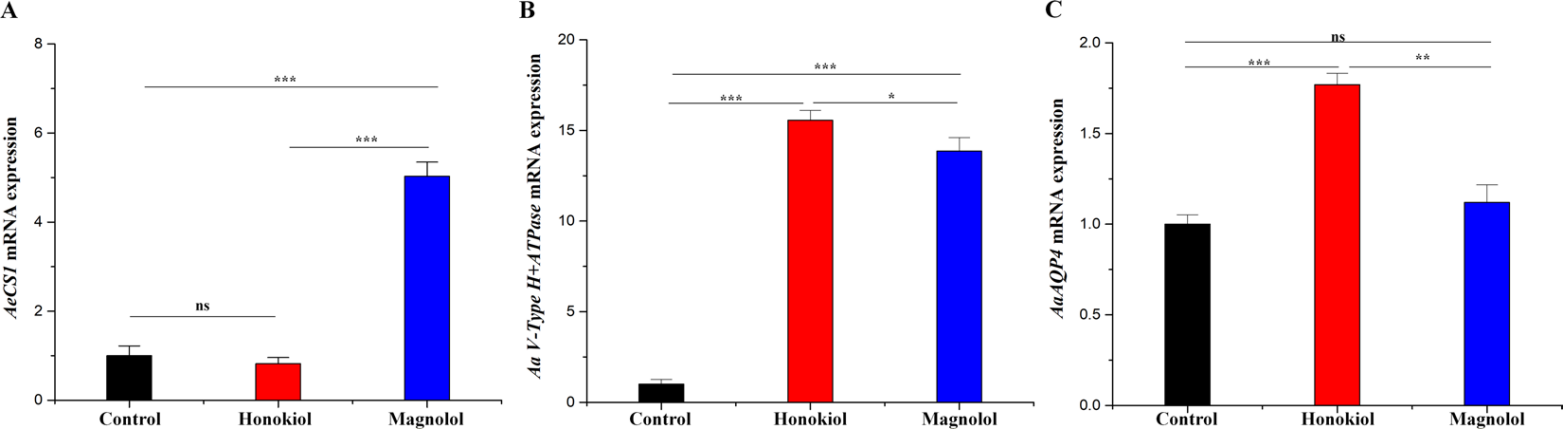
Effects of two neolignans on the expression levels of AeCS, AaV-type H^+^ ATPase, and AaAQP4 mRNA. Third-instar *Ae*. *aegypti* larvae were placed into paper cups containing a methanol–Triton X-100 solution in distilled water with an LC_50_ of honokiol (6.5 mg/L) or magnolol (25 mg/L) for 24 h. Total RNA was extracted from the anal gills (for AaAQP4 and AaV-type H^+^-ATPase) and midguts (for AeCS1) of 50 larvae. Real-time quantitative reverse transcription polymerase chain reaction was performed to determine the levels of AeCS1, AaV-type H^+^-ATPase, and AaAQP4 mRNA. Specific *AeCS1*, *AaV-type H*^+^*-ATPase*, and *AaAQP4*, and *Aarps7* coding sequence primers were used to amplify *AeCS1*, *AaV-type H*^+^*-ATPase*, *AaAQP4*, and *Aarps7* DNA, as described in the Methods section. **(A)** Midgut specific chitin synthase *AeCS1* gene expression was slightly inhibited in honokiol-treated larvae, whereas the gene expression level was significantly increased in magnolol-treated larvae. **(B)** *AaV-type H*^+^*-ATPase* gene expression was increased in honokiol- and magnolol-treated larvae compared to control larvae. **(C)** The *AaAQP4* gene expression level was significantly increased in honokiol-treated larval *Ae*. *aegypti* compared to control larvae. Magnolol did not affect the AaAQP4 mRNA expression level. The mRNA expression levels were normalized to constitutive expression of mRNA of the housekeeping gene *Aarps7* and analyzed by the 2^−ΔΔ*C*T^ method. Each bar represents the mean ± SE of duplicate samples run in three independent experiments (^***^P < 0.001; ^**^P < 0.01; ^*^P < 0.05; ns, no significant difference, using Bonferroni’s multiple comparisons test).

To investigate whether the two neolignans affected the transcription of *AeCS*, which is a midgut-specific chitin synthase gene^35^, we analyzed the AeCS1 mRNA levels of third-instar *Ae*. *aegypti* larvae using real-time quantitative reverse transcription polymerase chain reaction (qRT-PCR) (Fig. 9A). A reduction in the AeCS1 mRNA levels (82%) was observed in *Ae*. *aegypti* larvae following treatment with honokiol (6.5 mg/L) compared to the control larvae. However, the AeCS mRNA level was increased, by up to 503% of the control levels, following treatment with magnolol (25 mg/L).

The mRNA expression levels of AaV-type H^+^-ATPase, which is involved in the Na^+^, Cl^−^ and K^+^ uptake co-transport process^36,37^, and a putative AQP^38^ in the anal gills were likewise evaluated in third-instar *Ae*. *aegypti* larvae following treatment with honokiol (6.5 mg/L) or magnolol (25 mg/L). The AaV-type H^+^-ATPase mRNA expression levels in larvae treated with honokiol and magnolol, respectively, were approximately 16 and 14 fold higher than the control group (Fig. 9B). The AaAQP4 mRNA expression levels in larvae treated with honokiol were approximately 18 fold higher than the control group (Fig. 9C). By contrast, magnolol treatment did not affect the AaAQP4 mRNA expression levels.

## Discussion

Plant SMs, such as alkaloids, phenols and terpenoids, either alone or in combination, contribute to mosquito larvicidal activity. Selective plant preparations can be developed into mosquito larvicidal products that are suitable for IMM and are of great interest because they are target-specific, biodegrade to nontoxic products, have few harmful effects on nontarget organisms, and are environmentally nonpersistent^19–22^. These potential larvicidal products can be applied to habitats of mosquito larvae in the same manner as conventional larvicides. Certain plant preparations are toxic to different mosquito species larvae^19–22^ and have therefore been proposed to be alternatives to conventional larvicides. Active larvicidal constituents (LC_50_ < 50 mg/L^39^) derived from plants include alkaloids (e.g., retrofractamide A and three isobutylamides^40^, 0.004–0.86 mg/L), terpenoids (e.g., 9-oxoneoprocurcumenol and neoprocurcumenol^41^, 5.81 and 13.69 mg/L), coumarins (e.g., imperatorin and eight coumarins^26^, 2.88–44.39 mg/L), flavonoids (e.g., karanjin, karanjachromene, pongamol, and pongarotene^42^, 14.61–37.61 mg/L), phenylpropanoids (e.g., ethyl *p*-methoxycinnamate^43^, 12.3–20.7 mg/L), lignans (e.g., (−)-asarinin^25^, 10.49–16.49 mg/L), neolignans (e.g., burchellin^44^, 15.5 mg/L), cyanogenic glycosides (e.g., dhurrin^45^, 12 mg/L), lactones (e.g., butenolides 1 and 2^46^, 0.41 and 0.47 mg/L), acetylenic alcohols (e.g., falcarinol and falcarindiol^47^, 3.49 and 6.51 mg/L), and fatty acids (e.g., oleic acid and palmitic acid^42^, 18.07–18.45 and 34.50–42.96 mg/L). Kiran *et al*.^48^ considered compounds with an LC_50_ < 100 mg/L as having a significant larvicidal effect.

In the current study, we used a contact mortality bioassay to identify mosquito larvicidal constituents from *M*. *denudata* seed extracts. The active constituents were determined to be the saturated fatty acid palmitic acid (**1**), the unsaturated fatty acid linoleic acid (**2**), and the neolignan honokiol (**3**). Interpretations of the proton and carbon signals of compounds **1**, **2**, and **3** were largely consistent with the findings of Perumalsamy *et al*.^42^, Ragona *et al*.^49^, and Agrawal & Thakur^50^, respectively. The LC_50_ values of honokiol, linoleic acid, and palmitic acid were between 6.13 and 7.37 mg/L, between 6.97 and 7.50 mg/L, and between 32.56 and 47.58 mg/L, respectively, against larvae of four mosquito species (*Cx*. *pipiens pallens*, *Ae*. *aegypti*, *Ae*. *albopictus*, and *An. sinensis*). The LC_50_ values of the natural compounds described above were between 0.004 and 42.96 mg/L. Honokiol and linoleic acid were highly effective against three insecticide-susceptible *Cx. pipiens pallens*, *Ae. aegypti*, and *Ae. albopictus* larvae as well as *An. sinensis* larvae resistant to deltamethrin, temephos, and fenthion^31^. This current finding indicates that materials derived from *M*. *denudata* seeds could be promising naturally occurring mosquito larvicides that are novel and effective against wild mosquito populations in the field. Honokiol shows no toxicity or serious adverse effects in animal models^51^. Moreover, a honokiol microemulsion has been shown to be nontoxic up to 500 µg/kg body weight, although it was associated with irritation to the vasculature of the injection site^52^.

QSAR analyses of phytochemicals against larval mosquitoes are common^26,31,40,42,43,53,54^. Not only can QSAR information contribute to the search for additional compounds with higher activities but it can also promote greater understanding of the mechanism of larvicidal action of phytochemicals as described by Dias and Moraes^53^. The presence of lipophilic groups in aromatic rings or in hydroxyls results in increased toxicity to larval *Ae*. *aegypti*, whereas the presence of hydroxyls in aromatic or aliphatic rings results in decreased toxicity^54^. For example, phenol (LC_50_ 194 mg/L) was less toxic than compounds with lipophilic groups, such as CH chains outside a phenyl ring (e.g., carvacrol and thymol, LC_50_ 69 and 81 mg/L), against larval *Ae*. *aegypti*. Wang *et al*.^26^ studied the toxicity of six simple coumarins and seven furanocoumarins against third-instar *Cx*. *pipiens pallens* and *Ae*. *aegypti* larvae. They reported that the chemical structure, alkoxy substitution, and length of the alkoxy side chain at the C8 position of the coumarin nucleus were required for toxicity. However, the MW, hydrophobicity, and MR parameters were negatively correlated with the observed coumarin toxicity. The *N*-isobutyiamine moiety in isobutylamide alkaloids (pellitorine, guineensine, pipercide, and retrofractamide A) was found to play a crucial role in the mosquito larvicidal activity^40^.

In the current study, *p*-ethylphenol, *o*-eugenol, methoxyeugenol, *p*,*p*′-biphenol, and honokiol were the most toxic compounds against *Cx*. *pipiens pallens* and *Ae*. *aegypti* larvae. These compounds did not differ in toxicity from each other. Introduction of a functional group, such as ethyl, to phenol significantly increased the toxicity of the compounds. *o*-Eugenol and methoxyeugenol were significantly more toxic than either eugenol or isoeugenol. Introduction of a functional group, such as allyl or propenyl, to guaiacol significantly increased its toxicity. Eugenol or isoeugenol did not differ in toxicity and were more toxic than guaiacol. Honokiol was approximately 4 times more toxic than the structural isomer magnolol, which differs from honokiol only in the position of one hydroxyl group. Our findings indicate that phenolic compounds with an electron donor and/or bulky groups at the *ortho* or *para* positions were more toxic than phenolic compounds with an electron-withdrawing group or group at the *meta* position. The MW, hydrophobicity, and MR parameters appear not to be related to the observed phenol toxicity.

An investigation of the mechanisms of action and resistance mechanisms of botanical larvicides can provide useful information to develop efficient mosquito control alternatives with novel target sites and lower toxicities, as well as for determining the most appropriate formulations and delivery means to be adopted for their future commercialization and future resistance management^20,21,25^. The target sites and mechanisms underlying the insecticidal actions of several plant SMs have been well-documented by Isman^21^ and Pavela^22^. AChE is the main target site of mosquito larvicidal action of flavonoids (e.g., karanjin and pongarotene) and fatty acids (e.g., oleic acid and palmitic acid)^42^. The mechanism of the larvicidal action of the fatty acids elaidic acid and arachidic acid may involve interfering with the octopaminergic system, whereas linoleic acid and linolenic acid might act on both AChE and octopaminergic receptors^42^. Certain terpenoids inhibit AChE from houseflies, Madagascar roaches^55^, and head lice^56^. A relationship between insect toxicity and electric eel AChE inhibition by terpenoids has been reported^57^, whereas no direct correlation between the insecticidal and AChE inhibitory activities of terpenoids has been reported^42,55,56^. In addition, the major mechanisms of resistance to insecticides currently used to control mosquitoes include target site insensitivity, which reduces the sensitivity of sodium channels of the nervous system to pyrethroid insecticides, or sensitivity of the key enzyme AChE to OP and carbamate insecticides, as well as enhanced metabolic detoxification in various groups of insecticides^58^. Certain phytochemicals have been found to be highly effective against insecticide-resistant mosquitoes^25,26^, and these are likely to be useful in resistance management strategies. For example, α-asarone, (−)-asarinin, and pellitorine are effective against larvae of wild *Cx*. *pipiens pallens* and *An*. *sinensis*, which have high levels of resistance to the AChE inhibitors chlorpyrifos, fenitrothion, and fenthion; axonic nerve poisons α-cypermethrin and deltamethrin; and mitochondrial uncoupler chlorfenapyr^25^.

In the current study, a negative correlation (*r* = −0.609, *P* = 0.05) between *Ae*. *aegypti* larval toxicity and AChE inhibition by the 11 examined phenol compounds was observed. For example, guaiacol and caffeic acid were the most potent AChE inhibitors and were the least toxic larvicides. *o*-Eugenol, *p*,*p*′-biphenol, and *p*-ethylphenol were also highly toxic to *Ae*. *aegypti* larvae and were relatively weak AChE inhibitors. In addition, the isolated neolignan honokiol and its structural isomer magnolol moderately and strongly inhibited AChE, respectively, and caused a significant increase in cAMP levels. Our findings indicate that these two neolignans might act on both AChE and octopaminergic receptors, although which target site is more important remains to be elucidated. Furthermore, honokiol and magnolol are virtually equivalent in toxicity to both insecticide-susceptible *Ae*. *aegypti* and insecticide-resistant *An*. *sinensis* larvae, suggesting that these neolignans and the pyrethroid or OP do not share a common mode of action or elicit cross-resistance. Detailed tests are needed to understand the exact larvicidal mechanisms of honokiol and magnolol. The octopaminergic and gamma aminobutyric acid receptors have also been suggested to be novel target sites for several monoterpenoid essential oil constituents in *Helicoverpa armigera*^23^ and *Drosophila melanogaster*^24^, respectively.

Histopathological investigations indicated that the ME is the action site of plant SMs in *Rhodnius prolixus*^28^ and several aquatic dipteran larvae^29^. The ME has diverse functions, such as ionic and osmotic regulation^36^, lipid and carbohydrate storage^36,59,60^, midgut lumen pH control, digestive enzyme secretion, and nutrient absorption^61,62^. The histopathological effects differ qualitatively according to the localization of organs along the midgut and quantitatively according to the concentration of the material that was examined, duration of the treatment, and taxon^36^. The mosquito ME is composed of a single layer of polarized epithelial cells supported by an underlying basal lamina^63,64^. The tetranortriterpenoid azadirachtin has been reported to cause several of the initial effects of necrosis and is particularly associated with swelling of cell and organelles, vesiculation of membranes, and dilation of the rough endoplasmic reticulum in *Schistocerca gregaria* and *Locusta migratoria*^65^. It has also been reported that tannic acid, which is a highly water-soluble polyphenol, causes dramatic degenerative responses of the midgut through sequential epithelial disorganization in larval *Cx*. *pipiens*^29^. Tannins can bind to the lipid components of cell membranes^66^, leading to disruption of membrane integrity and/or function^29^.

The current microscopy analysis clearly indicated that the neolignans honokiol and magnolol caused histopathological alterations in the thorax, midgut, and anal gill regions of third-instar *Ae*. *aegypti* larvae. Honokiol-treated larval *Ae*. *aegypti* showed complete damage with an indistinct appearance of the whole body, particularly in the thorax and abdominal region segments, whereas magnolol-treated larvae showed unusual elongated digestive tracts with damaged interior tissues. Honokiol- or magnolol-treated larvae showed impaired anal gills with damaged outer cuticle layers of anal gill cells compared to control larvae, which showed well-developed anal gills and anal gill cells. In addition, the *AeCS* gene expression levels in control and honokiol-treated larvae did not differ significantly, whereas the gene expression level was significantly increased in magnolol-treated larvae. This finding suggests differences in the toxicity and mode of larvicidal action between honokiol and magnolol. It has been reported that the isobutylamide alkaloid pellitorine causes dramatic degenerative responses in the thorax and anterior and posterior midgut regions of larval *Ae*. *aegypti* by targeting ion transporting cells in the gastric caeca of the thorax region and epithelial cells of the anterior and posterior midgut regions^30^. Osmoregulation-related machineries, such as V-ATPase^37,67,68^, are highly expressed in the midguts of larval *Ae*. *aegypti*.

Larval mosquitoes rapidly respond as well as restore water and ion balance following stress. In larval *Ae*. *aegypti*, four anal gills surrounding the anal opening serve as the major sites for Na^+^, Cl^−^ and K^+^ uptake by H^+^-ATPase and Na^+^/K^+^-ATPase^37,69,70^. The osmotic uptake of water at the anal gills is the primary external site of ion uptake, normally contributing to 33% of the body weight gain per day^70^. Donini and O’Donnell^70^ measured the ion concentration gradients adjacent to the surface of the anal papillae of larval *Ae*. *aegypti* using the self-referencing ion-selective microelectrode technique. They confirmed that the anal gills of mosquito larvae serve as the major site for Na^+^, Cl^−^ and K^+^ uptake, complementing the role of the Malpighian tubules, an excretory and osmoregulatory organ, and the rectum, an organ that resorbs ions and some water from primary urine into the hemolymph. In addition, four putative AQP homologs are expressed in the Malpighian tubules, whereas two putative AQP homologs, AaAQP1b and AaAQP4, are expressed in the anal gills. In particular, AaAQP4 plays a crucial role in mediating water transport across the anal gill epithelia of the larval *Ae*. *aegypti*^38^. However, very little information is available with respect to the histopathological changes and gene expression patterns of AaV-type H^+^-ATPase and AaAQP4 induced by conventional insecticides or plant SMs on the anal gills of mosquito larvae. Perumalsamy *et al*.^30^ studied the histopathological and molecular effects of natural pellitorine on the larval ME and anal gills of *Ae*. *aegypti*. They reported that pellitorine inhibited the *AaAQP4* expression levels and suggested that this compound may disturb the Na^+^, Cl^−^ and K^+^ co-transport system, mainly by degeneration of anal gill cells and damaging the outer thick permeable cuticle membranes of the mosquito larvae. The alkaloid also caused slight decreases in the expression levels of the gene encoding the AaV-type H^+^-ATPase protein in the Na^+^, Cl^−^ and K^+^ ion co-transport system, despite significant effects on the gene expression level with pellitorine treatment^30^. Furthermore, expression of the genes encoding AaV-type H^+^-ATPase in the Na^+^, Cl^−^ and K^+^ ion transport system and AQP protein in the anal gills of larval *Ae*. *aegytpi* was determined in control and pellitorine-treated larvae^30^.

Our current study revealed that honokiol and magnolol caused increases in expression of the gene encoding the AaV-type H^+^-ATPase protein in the Na^+^, Cl^−^ and K^+^ ion co-transport system. In addition, honokiol treatment increased the *AaAQP4* expression level, whereas magnolol treatment did not affect this gene expression level. Our findings indicate that honokiol may disturb the Na^+^, Cl^−^ and K^+^ co-transport system, mainly by degeneration of anal gill cells and damaging the outer thick permeable cuticle membranes of larval *Ae*. *aegypti*. The ion exchange effects in the anal gills due to honokiol or magnolol treatment remain to be demonstrated, even though it has been reported that it is difficult to measure ion exchange *in situ* due to the morphological characteristics of mosquito larvae^71^. The finding that honokiol caused histopathological alterations and increased expression of AaV-type H^+^-ATPase and AQP may contribute to a better understanding of the mode of larvicidal action of this neolignan against *Ae*. *aegypti*. In addition, our findings, along with previous studies^30^, indicate that the mode of larvicidal action of honokiol might be different from that of the alkaloid pellitorine.

In conclusion, honokiol caused degenerative responses in the cell organelles of the thorax, midgut regions, and anal gills, possibly by targeting the osmoregulation system. *Magnolia denudata* seed-derived products may be useful as novel larvicides with specific target sites for controlling mosquito populations, particularly in light of their activity against insecticide-resistant mosquito larvae. *Magnolia denudata* is a fast-growing deciduous tree with the potential for efficient seed production and represents a potential source of a seed extract that can be used as an eco-product. Further research is needed regarding the practical applications of plant-derived preparations as novel mosquito larvicides to establish their safety profiles in humans, although *M*. *denudata* seed products have been widely used, as a carminative and diaphoretic^72^. In addition, the effects of these seed products on nontarget aquatic organisms, including larvivorous fishes; as biological control agents for mosquitoes^73^; and on the aquatic environment need to be established. Additional detailed tests are needed to understand how best to improve the larvicidal potency and stability of the compounds isolated from *M*. *denudata* for eventual product development.

## Methods

### Instrumental analysis

^1^H and ^13^C NMR spectra were recorded in deuterated chloroform on an AVANCE 600 spectrometer (Bruker, Rheinspettem, Germany) at 600 and 150 MHz, respectively, using tetramethylsilane as an internal standard. The chemical shifts are given in δ (ppm). The DEPT spectra were acquired using Bruker software. The UV spectra were obtained in ethanol or methanol on a UVICON 933/934 spectrophotometer (Kontron, Milan, Italy) and mass spectra on a JMS-DX 303 spectrometer (Jeol, Tokyo, Japan). Silica gel 60 (0.063–0.2 mm) (Merck, Darmstadt, Germany) was used for column chromatography. Merck precoated silica gel plates (Kieselgel 60 F_254_) were used for analytical thin-layer chromatography (TLC). An Isolera One medium-pressure liquid chromatograph (Biotage, Uppsala, Sweden) and Agilent 1200 high-performance liquid chromatograph (Agilent, Santa Clara, CA, USA) were used to isolate the active constituents.

### Materials

Three constituents, palmitic acid, linoleic acid and honokiol, were identified in this study, and their pure organic compounds were purchased from Sigma-Aldrich (St. Louis, MO, USA). Magnolol, which is a structural isomer of honokiol, and nine commercially available pure organic phenol compounds are listed in Table 8 along with their purities and were used in this study for QSAR. These phenol compounds were purchased from Sigma-Aldrich. For the QSAR analysis, the values of MW, hydrophobic parameter (log *P*), and steric effects for these compounds were obtained from ChemDraw Ultra 10.0 (Cambridge Soft Corporation, Cambridge, MA, USA) and recorded in Table 8. The MR was used as the parameter to describe steric effects. The structures of these phenol compounds are shown in Fig. 2. The OPs temephos (97.3% purity) and analytical dichlorvos were purchased from Sigma-Aldrich. Acetylthiocholine iodide (ATChI), 5,5′-dithio-bis(2-nitrobenzoate) (DTNB), and octopamine were supplied by Sigma-Aldrich. The cAMP Biotrak Enzymeimmunoassay system and bovine serum albumin (BSA) were supplied by GE Healthcare (Little Chalfont, Buckinghamshire, UK) and Sigma-Aldrich, respectively. Horseradish peroxidase (HRP)-labeled cAMP was purchased from R&D Systems (Minneapolis, MN, USA). All of the other chemicals and reagents used in this study were of reagent-grade quality and are available commercially.

**Table 8:** Values of physical parameters of 11 phenol compounds examined in this study.

Compound	Molecular weight (g/mol)	log *P*	Molecular refraction	Purity (%)
Phenol	94.11	1.48	28.1	≥99
*p*-Ethylphenol	122.17	2.42	37.7	99
Guaiacol	124.14	1.19	34.8	98
*o*-Eugenol	148.21	2.96	46.9	98
Eugenol	164.20	2.20	48.7	99
Isoeugenol	164.20	2.45	50.7	98
Caffeic acid	180.16	1.42	47.5	≥98
*p*,*p′*-Biphenol	186.21	2.42	54.6	97
Methoxyeugenol	194.23	1.79	55.4	≥95
Honokiol	266.33	4.20	82.4	≥98
Magnolol	266.33	3.94	82.4	≥98

### Plant material

Fresh seeds of *M*. *denudata* were collected from the garden at Huazhong Agriculture University (30°34′60′′N, 114°16′0′′E) (Wuhan, Hubei, China) in August 2011. The plant was identified by a certified botanical taxonomist. A voucher specimen (MD-SD-01) was deposited in the Research Institute of Agriculture and Life Sciences at Seoul National University.

### Mosquitoes

The stock cultures of *Cx*. *pipiens pallens* and *Ae*. *aegypti* were maintained in the laboratory without exposure to any known insecticide^25^. Engorged *Ae*. *albopictus* and *Anopheles* females were collected from bamboo forest near a village in Jeonju (Jeonbuk, Republic of Korea (ROK)) and rice paddy fields and cowsheds at Osong (Chungbuk, ROK), respectively, in August 2011 using black light FL-6w traps (Shinyoung, Seoul, ROK) and a D-CELL collecting aspirator (Gemplers, Janesville, WI, USA). These mosquito species were separately maintained in temperature-controlled insect rearing rooms to prevent cross-contamination. Females were placed individually in paper cups (270 mL) lined with filter paper and filled with 150 mL distilled water (DW). Larvae and adults were reared in plastic trays and nylon cages, respectively, under the same conditions as those described previously^74^. All stages were maintained at 27 ± 1 °C and 65–75% relative humidity under a 16:8 h light:dark cycle. Species identification for *Anopheles* females based on PCR confirmed that females from the wild collections were *An. sinensis*^31^. *Aedes albopictus* and *An. sinensis* mosquitoes were reared for 4–5 generations to ensure sufficient numbers for testing. Larvae of *An*. *sinensis* showed extremely high level of resistance to deltamethrin (relative susceptibility ratio (RSR), 611) and moderate levels of resistance to temephos (RSR, 29) and fenthion (RSR, 11) compared with *Ae*. *aegypti* larvae^31^. Larvae of *Ae*. *albopictus* showed similar susceptibilities to deltamethrin, temephos, and fenthion as *Ae*. *aegypti* larvae^31^.

### Bioassay-guided fractionation and isolation

Seeds (1.5 kg) of *M*. *denudata* were pulverized, extracted with methanol (5 × 3 L) at room temperature for 2 days, and filtered. The combined filtrate was concentrated by rotary evaporation at 40 °C to yield approximately 266.4 g of a dark brown tar. The extract (20 g) was sequentially partitioned into hexane- (3.11 g), chloroform- (5.10 g), ethyl acetate- (6.09 g), butanol- (1.53 g), and water-soluble (4.17 g) portions for subsequent bioassays. This fractionation procedure was repeated 12 times. The organic solvent-soluble portions were concentrated at 40 °C as described above, and the water-soluble portion was freeze-dried. To isolate the active constituents, 10–100 mg/L of each *M*. *denudata* seed-derived fraction was tested in a contact mortality bioassay as described by Perumalsamy *et al*.^25^.

**Figure 10:**
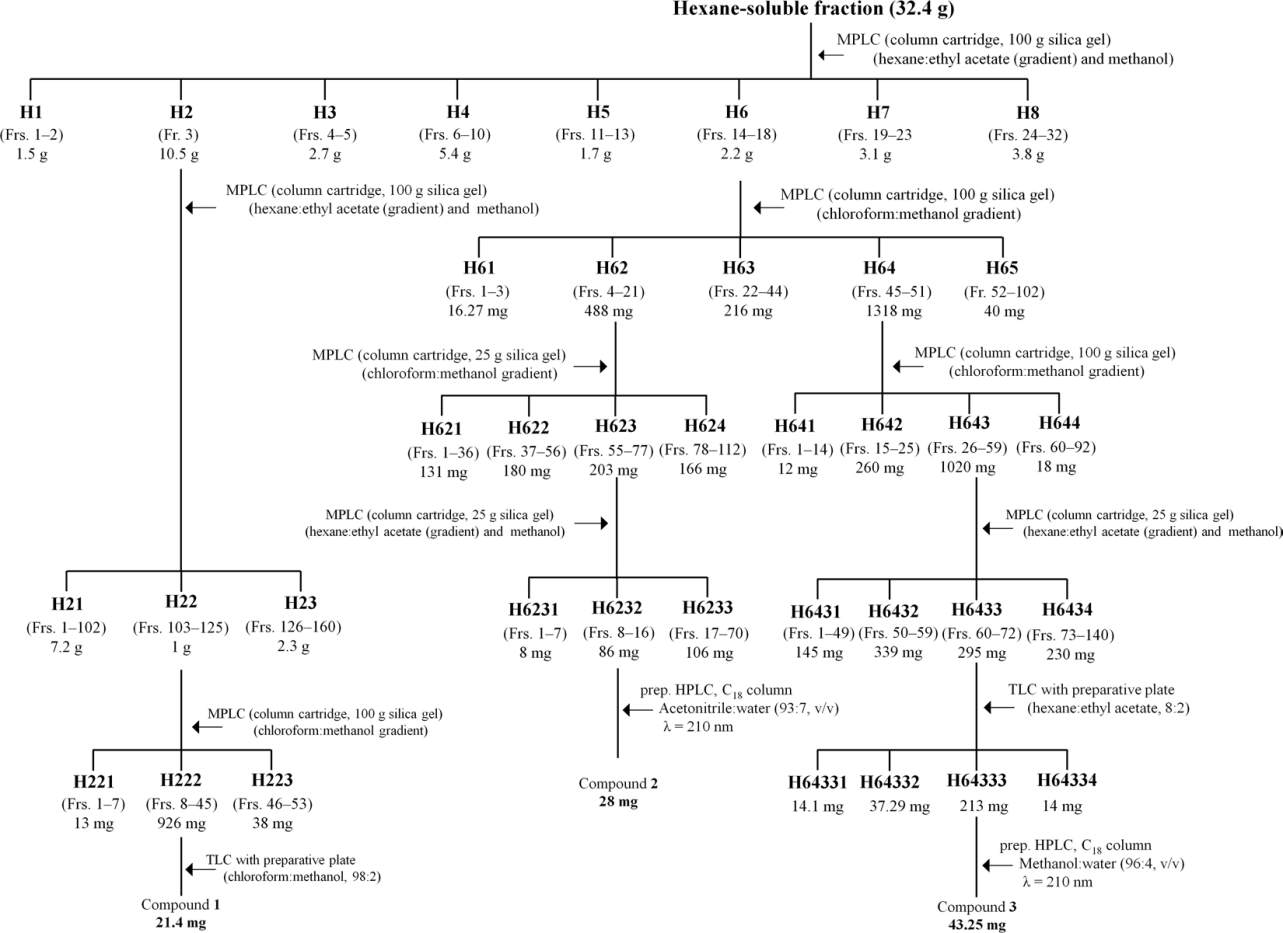
Procedures to isolate the mosquito larvicidal constituents. The *Magnolia denudata* seed methanol extract was sequentially partitioned into hexane-, chloroform-, ethyl acetate-, butanol-, and water-soluble portions. The hexane-soluble fraction was the most biologically active fraction, and medium-pressure liquid chromatography and high-performance liquid chromatography were performed. Each fraction (10–100 mg/L) was tested in a contact mortality bioassay against third-instar *Culex pipiens pallens* and *Aedes agypti* larvae to isolate the active constituents from the fraction.

The hexane-soluble fraction (32.4 g) was the most biologically active fraction (Table 1), and medium-pressure liquid chromatography (MPLC) was performed using an Isolera apparatus equipped with a UV detector at 254 and 365 nm and SNAP column cartridge (100 g silica gel) with a column volume of 132 mL (Fig. 10). Separation was achieved with a gradient of hexane and ethyl acetate (100:0, 98:2, 90:10, 80:20, 70:30, 60:40, 50:50, 60:40, and 0:100 by volume), and then, elution with methanol (1 L) was performed at a flow rate of 30 mL/min to provide 42 fractions (each approximately 100 mL). The column fractions were monitored by TLC on silica gel plates developed with a chloroform and methanol (8:2 by volume) mobile phase. Fractions with similar *R*_f_ values on the TLC plates were pooled. The spots were detected by spraying the plate with 2% sulfuric acid and then heating the samples on a hot plate. Active fractions 3 (H2) and 14–18 (H6) were obtained. Fraction H2 was separated by MPLC with a UV detector and column cartridge (100 g silica gel) through elution with a gradient of hexane and ethyl acetate (98:2, 97:3, 96:4, 95:5, 90:10, 87:13, 83:17, 80:20, 73:17, 65:35, 60:40, and 50:50 by volume), followed by a final elution with methanol (1 L) at a flow rate of 50 mL/min to provide 233 fractions (each approximately 22 mL). The column fractions were monitored by TLC on silica gel plates as described above. Active fractions 103–125 (H22) were pooled and separated by MPLC with a UV detector at 254 and 280 nm and column cartridge (25 g silica gel) with a column volume of 33 mL by elution with a gradient of chloroform and methanol (100:0, 99:1, 98:2, 97:3, 80:20, and 0:100 by volume) at a flow rate of 15 mL/min to provide 70 fractions (each approximately 20 mL). Fractions 8–45 (H222) was purified by preparative TLC (chloroform:methanol, 98:2 by volume) to yield compound **1** (21.4 mg). Another active fraction, H6, was separated by MPLC with a UV detector at 254 and 360 nm and column cartridge (100 g silica gel) by elution with a gradient of chloroform and methanol (100:0, 99:1, 98:2, 97:3, 96:4, 93:7, 90:10, 89:11, 60:40, and 0:100 by volume) at a flow rate of 25 mL/min to provide 204 fractions (each approximately 22 mL). The column fractions were monitored by TLC on silica gel plates developed with a hexane and ethyl acetate (9:1 by volume) mobile phase. Active fractions 4–21 (H62) and 45–51 (H64) were obtained. Fraction H62 was separated by MPLC with a UV detector at 254 and 280 nm and column cartridge (100 g silica gel) by elution with a gradient of hexane and ethyl acetate (96:4, 94:6, 90:10, 85:15, 80:20, 77:23, 73:27, 60:40, and 40:60 by volume), followed by a final elution with methanol (350 mL) at a flow rate of 40 mL/min to provide 110 fractions (each approximately 22 mL). The column fractions were monitored by TLC (hexane:ethyl acetate, 8:2 by volume). Active fractions 57–77 (H623) were pooled and separated by MPLC with a UV detector at 254 and 365 nm and column cartridge (25 g silica gel) by elution with a gradient of hexane and ethyl acetate (100:0, 96:4, 90:10, 85:15, 82:18, 81:19, 75:25, 70:30, 57:43, and 20:80 by volume), followed by a final elution with methanol (80 mL) at a flow rate of 25 mL/min to afford 68 fractions (each approximately 22 mL). Preparative high-performance liquid chromatography (HPLC) was performed to separate the constituents from active fractions 8–16 (H6232) from H623 with a 7.8 mm i.d. × 300 mm µBondapak C18 column (Waters, Milford, MA, USA) and mobile phase of acetonitrile and water (93:7 by volume) at a flow rate of 1 mL/min. Chromatographic separation was monitored using a UV detector at 210 nm. Finally, active constituent **2** (28 mg) was isolated at a retention time of 19.81 min. Fraction H64 was separated by MPLC as described above. The column fractions were monitored by TLC on silica gel plates developed with hexane and ethyl acetate (8:2 by volume). Active fractions 26–59 (H643) were pooled and separated by MPLC with a UV detector at 254 and 365 nm and column cartridge (100 g silica gel) by elution with a gradient of chloroform and ethyl acetate (100:0, 99:1, 98:2, 97:3 and 96:4 by volume), followed by a final elution with methanol (300 mL) at a flow rate of 40 mL/min to afford 147 fractions (each approximately 22 mL). Fractions 60–72 (H6433) were pooled and purified by preparative TLC (chloroform:ethyl acetate, 8:2 by volume) to provide four fractions. A preparative HPLC was performed to separate the constituents from active fractions H64333 from H6433 as described above. Finally, potent active constituent **3** (43.25 mg) was isolated at a retention time of 13.57 min.

### Bioassay

A contact mortality bioassay^25^ was used to evaluate the toxicity of all compounds against third-instar larvae of the four mosquito species. Each test compound in methanol was suspended in DW with Triton X-100 (20 µL/L). Groups of 20 mosquito larvae were separately placed into paper cups (270 mL) containing each compound solution (250 mL). Temephos served as a positive control and was used in a similar manner. Negative controls consisted of a methanol–Triton X-100 solution in DW. As judged by the preliminary test results, the toxicity of each test compound and insecticide was determined with four to six concentrations ranging from 0.1 to 400 mg/L and 0.001 to 0.1 mg/L, respectively.

Treated and control (methanol–Triton X-100 solution only) larvae were maintained under the same conditions as those used for colony maintenance without providing food. Larval mortalities were determined 24 h post-treatment. A larva was considered dead if it did not move when it was prodded with a fine wooden dowel. All treatments were performed three times using 20 larvae per replicate.

### Acetylcholinesterase inhibition assay

The *in vitro* AChE inhibitory activity of the test compounds was assessed according to the method of Perumalsamy *et al*.^42^. Third-instar *Ae*. *aegypti* larvae were used in all experiments. Whole bodies of 50 larvae were homogenized in 1 mL of ice-cold 0.1 M phosphate buffer (pH 8.0) using a glass tissue homogenizer. After filtering through cheesecloth, the homogenate was centrifuged at 1,000 × *g* at 4 °C for 5 min. The supernatant was used directly as the enzyme source for AChE. The protein levels were determined using the Bradford Protein Assay Kit (Bio-Rad Laboratories, Hercules, CA, USA) using BSA as the standard. The reaction mixture consisted of 50 µL of the crude enzyme preparation (4.3–4.8 µg protein equivalents), 150 µL of 0.1 M phosphate buffer, 20 µL of 3 mM DTNB in phosphate buffer (pH 7.0), and 1 µL of various concentrations of each test compound in ethanol. The reaction mixture was incubated at 30 °C for 5 min, and 20 µL of 32 mM ATChI was then added to the mixture. After incubation for 30 min, the reaction was terminated by adding 20 µL of 5 mM eserine salicylate. Absorbance was recorded at 412 nm using a VersaMax microplate reader (Molecular Devices, Sunnyvale, CA, USA). The OP dichlorvos served as a positive control and was used in a similar manner. The results are expressed as the means ± standard errors (SEs) of triplicate samples from three independent experiments.

### Determination of cyclic AMP levels

The method of Perumalsamy *et al*.^42^ was used to assess the *in vitro* octopamine-sensitive adenylate cyclase activity of the test compounds. In brief, whole bodies of 75 *Ae*. *aegypti* larvae (106.2 mg) were homogenized in 500 µL of 2 mM Tris-maleate buffer (pH 7.4) containing 0.8 mM ethylene glycol tetraacetic acid (EGTA). The adenylate cyclase activity was determined using the cAMP Biotrak Enzymeimmunoassay system according to the manufacturer’s protocol. The assay was conducted in a total volume of 100 µL containing 80 mM Tris-maleate buffer, 5 mM theophylline, 2 mM MgSO_4_, 0.5 mM adenosine triphosphate (ATP), 0.2 mM EGTA, 50 µL of whole-body homogenate (equivalent to 4.12 µg protein), and 1 µL of the test compounds in Tris-maleate buffer containing 0.2% ethanol. After incubation at 20 °C for 5 min, the reaction was initiated by addition of ATP. Incubation was performed at 30 °C for 3 min in a shaking water bath. The reaction was stopped by boiling for 2 min, and then, the assay tube was cooled and centrifuged at 8,000 × *g* for 10 min. Fifty microliters of the supernatant were assayed for cAMP levels.

Polystyrene microplates (1 strip of 8 wells) coated with a goat anti-mouse polyclonal antibody were used. Fifty microliters of a mouse monoclonal antibody solution were added to each well except for the blank wells (or the nonspecific binding wells). The wells covered with the adhesive strip were incubated at 25 °C for 1 h in a shaking incubator (480 rpm). After washing with 400 µL of wash buffer four times, 50 μL of the two neolignan samples for cAMP determination and cAMP standard were added to wells. Control, blank (nonspecific binding), and zero standard wells were supplemented with 50 μL of a diluent RD5-55 buffer. Fifty microliters of HRP-labeled cAMP were then added to the wells. The plate was covered with a new adhesive strip and incubated at 25 °C for 2 h on the shaker. After an additional wash as described above, 200 μL of a substrate solution (equal volumes of stabilized hydrogen peroxide and stabilized chromogen) was added to each well and the test plate was incubated at 25 °C for 30 min on the benchtop in darkness. Finally, the reaction was terminated by addition of 100 μL of 2 *N* sulfuric acid to each well. The optical densities at 450 and 540 nm were determined using the microplate reader described above. The readings at 540 nm were subtracted from the readings at 450 nm. The cAMP concentrations are expressed as nmol/µg protein. The results are expressed as the means ± SEs of triplicate samples from three independent experiments.

### Light microscopy analysis

Third-instar *Ae*. *aegypti* larvae were placed into paper cups (270 mL) containing a methanol–Triton X-100 solution (250 mL) in DW with an LC_50_ of honokiol (6.5 mg/L) or magnolol (25 mg/L) as described previously^25^. At 24 h post-treatment, the untreated and treated larvae were placed on a microscope slide at room temperature for light microscopy. Morphological observations were made with an EZ4 HD stereo microscope (35×) equipped with an Integrated 3.0 Mega-Pixel CMOS camera (Leica Microsystems, Heerbrugg, Switzerland). All experiments were performed in duplicate, with 20 mosquito larvae used in each replicate. More than 10 live larvae from the control and treated groups were randomly collected and used for analysis.

### Histologic analysis by Carson’s trichome staining

Third-instar *Ae*. *aegypti* larvae were treated with an LC_50_ of honokiol (6.5 mg/L) or magnolol (25 mg/L) in all experiments as described above. At 24 h post-treatment, *Ae*. *aegypti* larvae untreated and treated with honokiol or magnolol were immediately fixed in Bouin’s fluid^75^ at 4 °C for 24 h. Larvae were then dehydrated in an ethanol-tetrahydrofuran-xylene series and embedded in Paraplast X-tra (Sigma-Aldrich). The embedded preparations of the larvae were sectioned at a 5 μm thickness using a Microm HM 340E rotary microtome (Thermo Scientific, Walldorf, Germany). The sections were dried at 40 °C overnight, subsequently deparaffinized with CitriSolv (Fisher Scientific, Fair Lawn, NJ, USA), and rehydrated with a series of ethanol in phosphate-buffered saline (PBS) solutions^30^. The rehydrated sections were stained in Weigert’s iron hematoxylin for 30 s, followed by Carson’s trichrome staining procedure^76^. This staining protocol stained the columnar and goblet cells of the midgut blue and red, respectively. These sections were then dehydrated, cleared in xylene, and mounted in EMS Permount (Electron Microscopy Sciences, Hatfield, PA, USA). Images were observed and captured using a DMIL LED microscope (Leica Microsystems, Wetzlar, Germany) equipped with a Leica MC 170 HD. Observations were taken of 15 larvae under the microscope.

### Transmission electron microscopy analysis

Third-instar *Ae*. *aegypti* larvae were treated with an LC_50_ of honokiol (6.5 mg/L) or magnolol (25 mg/L) in all experiments as described above. At 24 h post-treatment, the midgut and anal gills of the untreated and treated *Ae*. *aegypti* larvae were fixed in Karnovsky’s fixative (2% (v/v) glutaraldehyde and 2% (v/v) paraformaldehyde in 0.05 M sodium cacodylate buffer (pH 7.2)) at 4 °C in darkness for 2–4 h and were washed three times with the same buffer. The specimens were post-fixed with 1% (w/v) osmium tetroxide in the same buffer at 4 °C for 2 h and washed with DW three times. The post-fixed specimens were then dehydrated through a graded series of ethanol with increasing concentrations up to 100% for 15 min. The specimens were further treated with propylene oxide twice each for 15 min as a transitional fluid and embedded in Spurr’s resin^77^. Ultrathin sections (approximately 50 nm thickness) were cut with a UC6 ultramicrotome (Leica Microsystems, Wetzlar, Germany) and stained in 2% aqueous uranyl acetate for 7 min at room temperature as well as with Reynolds lead citrate^78^ for 7 min. The sections were mounted on copper grids, and micrographs were obtained from a CM120 transmission electron microscope (Philips Electronics, Amsterdam, Netherlands) at 80 kV. Images were captured using a MegaView III digital camera (Olympus-SIS, Lakewood, CO, USA). Observations were taken of 12 larvae via TEM.

### Real-time reverse transcription-polymerase chain reaction assay

Real-time qRT-PCR with SYBR Green dye was performed to determine whether honokiol and magnolol treatment affected the expression levels of three target genes, *AaAQP4*, *AaV-type H*^+^*-ATPase* and *AeCS1*, in *Ae*. *aegypti*. Third-instar *Ae*. *aegypti* larvae were placed into paper cups (270 mL) containing a methanol–Triton X-100 solution (250 mL) in DW with an LC_50_ of honokiol (6.5 mg/L) or magnolol (25 mg/L) for 24 h as described previously^25^. Total RNA was extracted from the anal gills (for AaAQP4 and AaV-type H^+^-ATPase) and midgut (for AeCS1) of 50 larvae using an RNeasy Mini Kit (Qiagen, Valencia, CA, USA) according to the manufacturer’s instructions. Residual genomic DNA was removed using RQ1 RNase-Free DNase (Promega, Fitchburg, WI, USA), and 1 μg of total RNA from each sample was used for complementary DNA (cDNA) synthesis with an oligo(dT) primer (Invitrogen, Grand Island, NY, USA) according to the protocol of the SuperScript III Reverse Transcriptase Kit. Real-time qRT-PCR was performed in 96-well plates using the StepOne Plus Real-Time PCR System (Applied Biosystems, Foster, CA, USA). Each reaction mixture consisted of 10 μL of the SYBR Green PCR Master Mix Kit (Applied Biosystems, Warrington, Cheshire, UK), 2 μL of the forward and reverse primers (10 pmol each), 1 μL of cDNA (25 ng) and diethylpyrocarbonate-treated water for a final volume of 20 μL. The oligonucleotide PCR primer pairs are listed in Table 9 and were purchased from Cosmo Genetech (Seoul). The cycling program included an initial hold at 95 °C for 5 min, followed by 35 cycles at 95 °C for 30 s, at 58 °C for 30 s, at 72 °C for 1 min, and an extension at 72 °C for 5 min using an Applied Bioscience Thermal Cycler. The mRNA expression levels of the target genes were normalized to the mRNA expression level of the housekeeping gene *Aarps7* and analyzed by the 2^−ΔΔ*C*T^ method using the Applied Biosystems StepOne Software v2.1 and DataAssist Software. The results are expressed as the means ± SEs of duplicate samples from three independent experiments.

**Table 9:** Primers used for real-time quantitative reverse transcription polymerase chain reaction in this study.

Gene	RefSeq ID	Forward primer and reverse primer	cDNA amplicon size
*Aarps7*	AY380336.1	5′-CTGGAGGATCTGGTCTTC-3′ 5′-GTGTTCAATGGTGGTCTG-3′	838
*AeCS1*	XM_001662150	5′-GGTCGCTGAGGATAGTGAAG-3′ 5′-CCGTGCTGGAGAGATGAAGTC	4767
*AaAQP4*	XM_001647996	5′-ATGCCACTGCTTGTCCCTAC-3′ 5′-TTTCCGAAATGACCTTGGAG-3′	786
*AaV-type H* ^+^ *-ATPase*	AF092934	5′-GTTGTTCTGGCTCTGCTGTTA-3′ 5′-GAGTGTTCTCGATAAGCCATAATC-3′	2538

### Data analysis

The concentration of the test compounds required to produce 50% AChE inhibitory activity (IC_50_) was determined using the SAS 9.13 program (Cary: SAS Institute; 2014). Concentration-mortality data were subjected to probit analysis using the SAS 9.13 program. The LC_50_ values for the treatments were considered significantly different from one another when the 95% confidence intervals (CIs) did not overlap. A compound that had an LC_50_ > 100 mg/L was considered to be ineffective as described by Kiran *et al*.^48^. Correlation coefficient analysis of the toxicities of compounds to third instar *Cx*. *pipiens pallens* and *Ae*. *aegypti* larvae was performed using their LC_50_ values and physical parameter (MW, MR, and log *P*) values for the test phenol compounds. One-way analysis of variance was used as indicated in the tables and figure legends. Statistical analyses were performed using the SAS 9.13 program. Multiple linear regression and correlation analysis were performed using the SPSS 24 program (Armonk: IBM Corp.; 2016).

## Electronic supplementary material

Supplementary Info

## Data Availability

All data are disclosed in the text or tables in the article. EI-MS, ^1^H NMR, ^13^C NMR, and DEPT spectra of compounds 1, 2, and 3 are provided as Additional files 1–3, 4–7, and 8–11, respectively.
