# Supplementary material for: Toxicity and possible mechanisms of action of honokiol from Magnolia denudata seeds against four mosquito species
Source: Sci Rep. 2019 Jan 23;9:411. doi: 10.1038/s41598-018-36558-y (PMC6344527; doi:10.1038/s41598-018-36558-y)
Supplement: Supplementary file 1 — Supplementary Info [file 41598_2018_36558_MOESM1_ESM.pdf]

Toxicity and possible mechanisms of action of honokiol from *Magnolia denudata* seeds against four mosquito species

Zhangqian Wang[1,2†], Haribalan Perumalsamy[3†], Xue Wang[4] and Young-Joon Ahn[1,3*]

[1]Department of Agricultural Biotechnology, Seoul National University, Seoul 08826, Republic of Korea

[2]Key Laboratory of Combinatorial Biosynthesis and Drug Discovery (Wuhan University), Ministry of Education, and Wuhan University School of Pharmaceutical Sciences, Wuhan 430071, Hubei, China

[3]Research Institute of Agriculture and Life Sciences, Seoul National University, Seoul 08826, Republic of Korea

[4]School of Pharmaceutical Science, Wenzhou Medical University, Wenzhou 325035, Zhejiang, China

[†] These authors contributed equally to this work

Running Head: Toxicity and mechanism of action of honokiol

[*] Correspondence: yjahn@snu.ac.kr

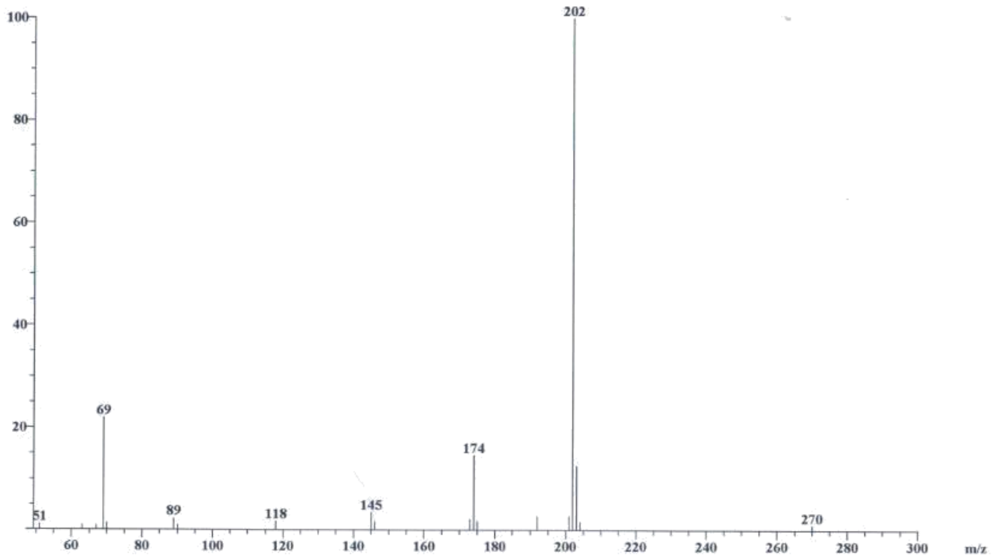

Figure S1. EI-MS (70 eV) spectrum of palmitic acid 1.

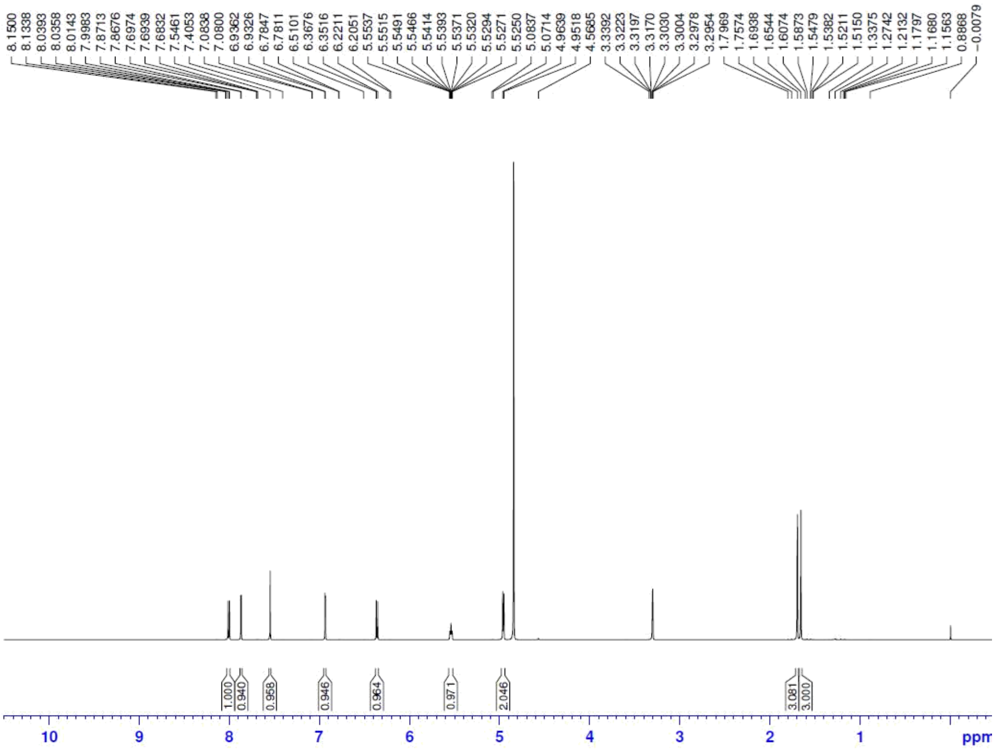

Figure S2. ^{1}**H NMR (CDCl$_3$, 600 MHz) spectrum of palmitic acid 1.**

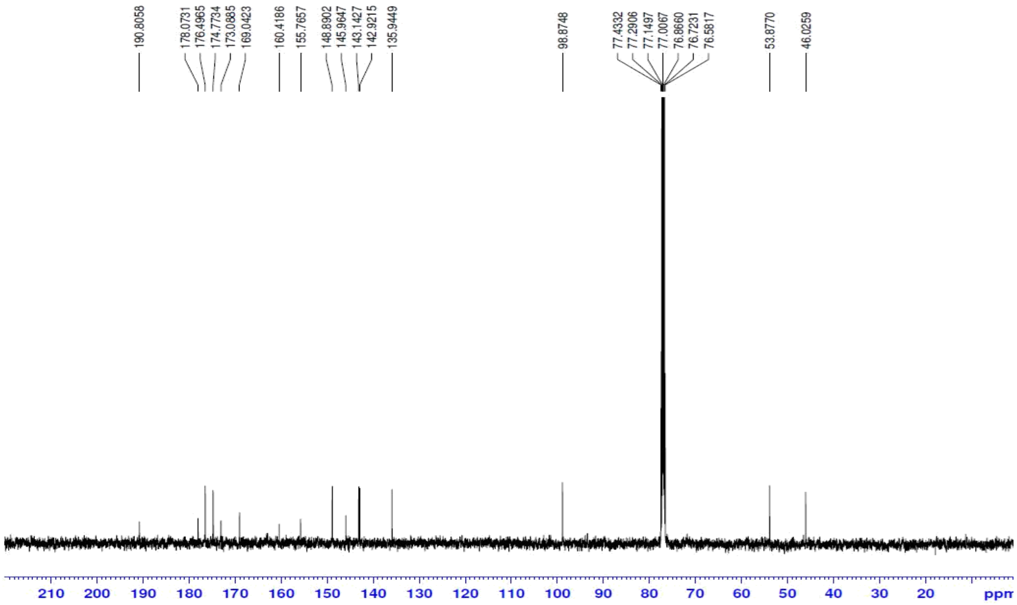

Figure S3. ^{13}C **NMR ($CDCl_3$, 150 MHz) spectrum of palmitic acid 1.**

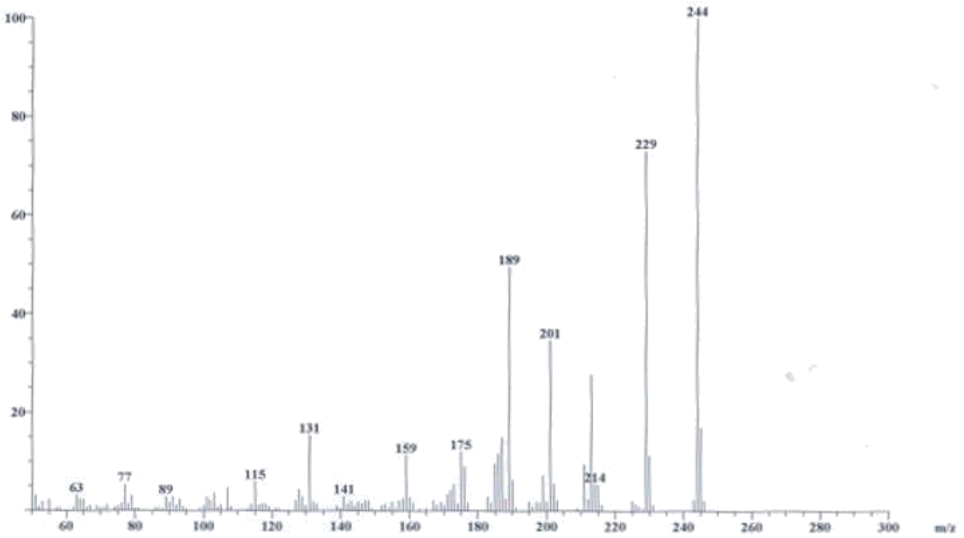

Figure S4. EI-MS (70 eV) spectrum of linoleic acid 2.

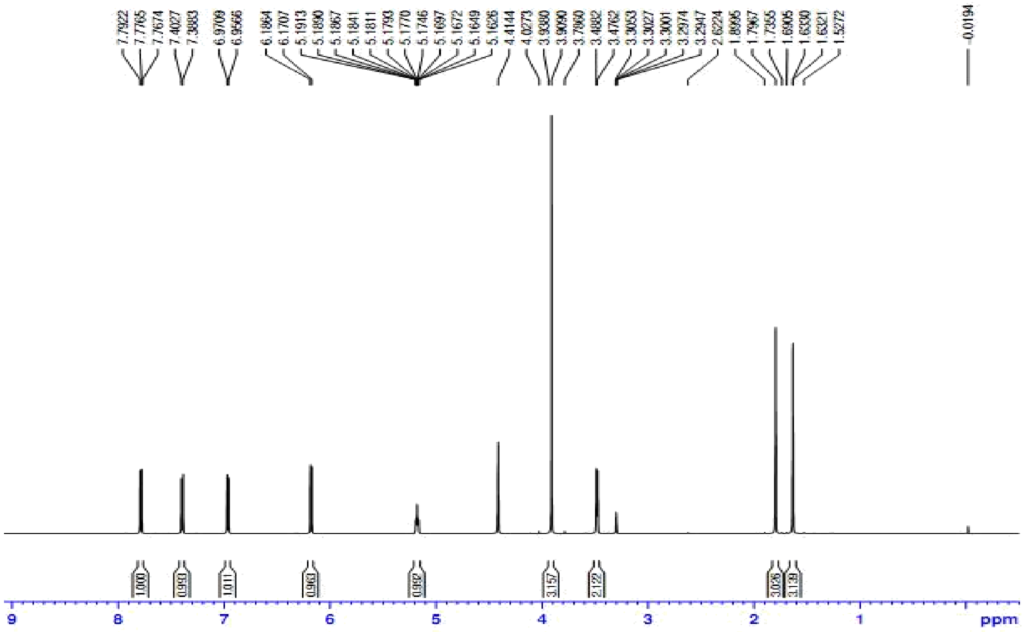

Figure S5. ^{1}**H NMR ($CDCl_3$, 600 MHz) spectrum of linoleic acid 2.**

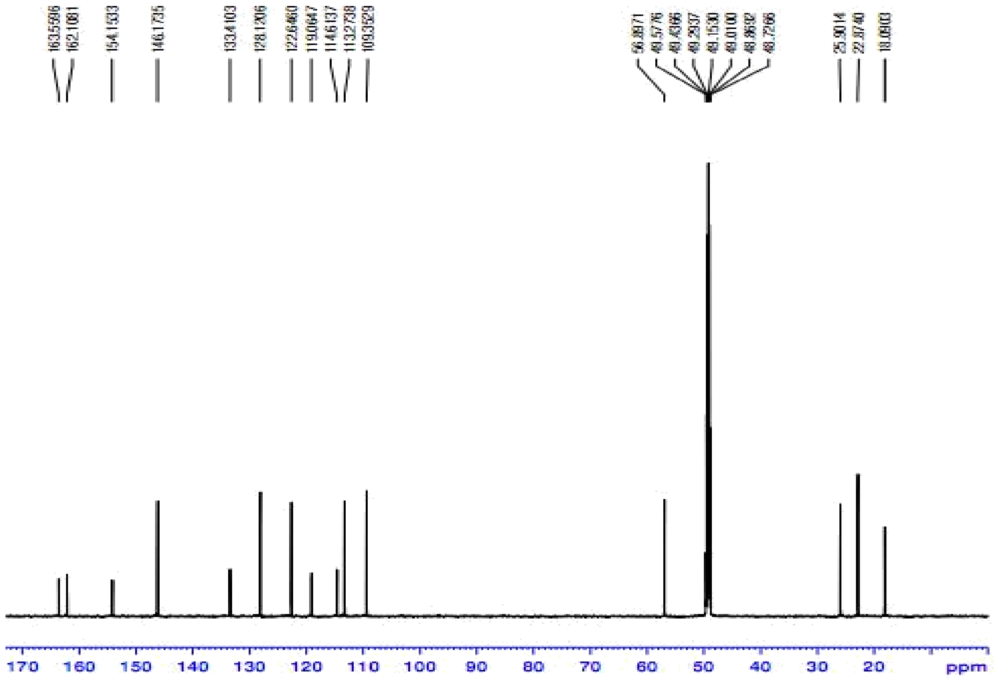

Figure S6. ^{13}C **NMR ($CDCl_3$, 150 MHz) spectrum of linoleic acid 2.**

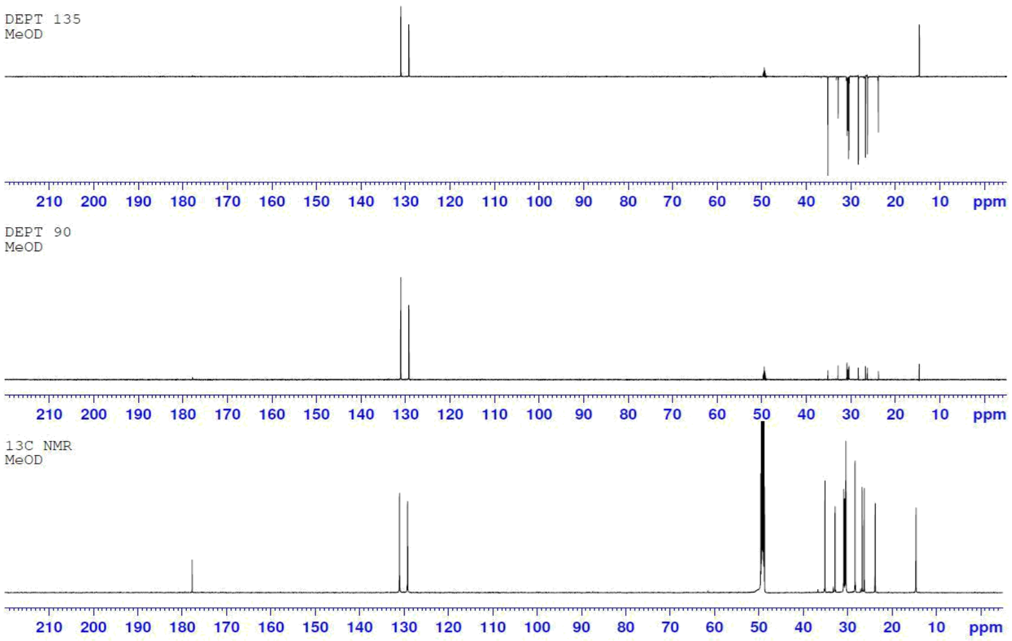

Figure S7. DEPT spectrum of linoleic acid 2.

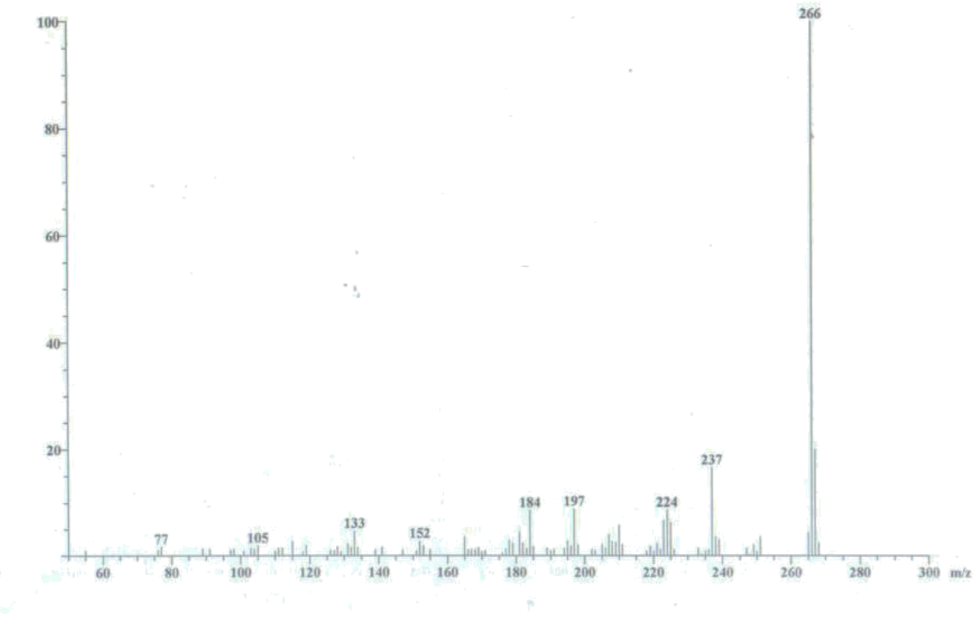

Figure S8. EI-MS (70 eV) spectrum of honokiol 3.

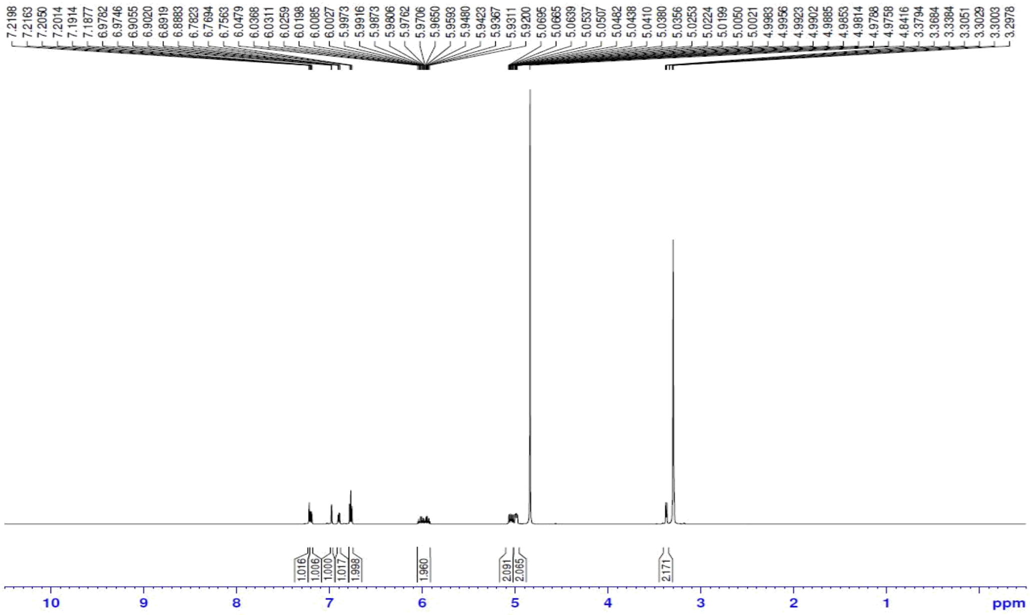

Figure S9. ^{1}H **NMR ($CDCl_3$, 600 MHz) spectrum of honokiol 3.**

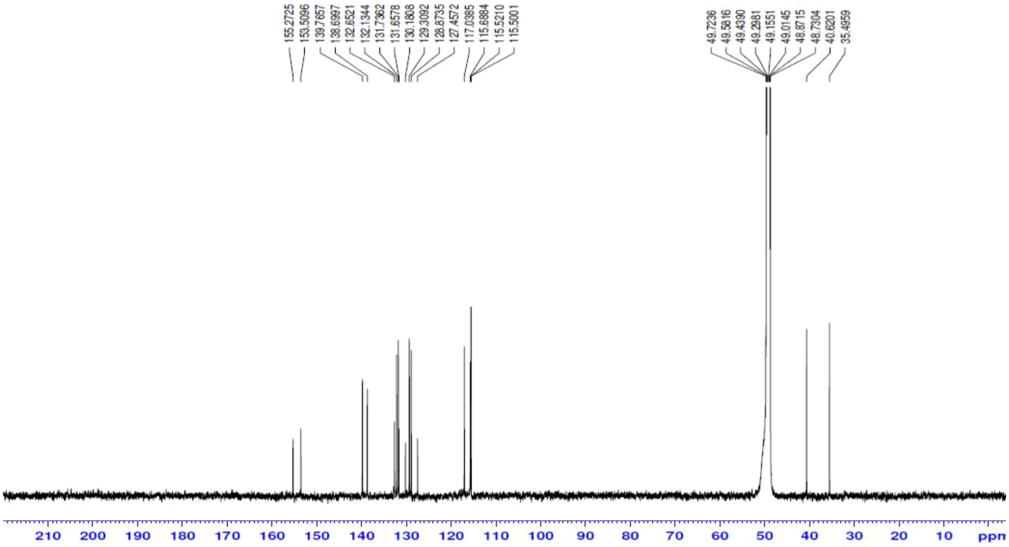

Figure S10. **^{13}C NMR ($CDCl_3$, 150 MHz) spectrum of honokiol 3.**

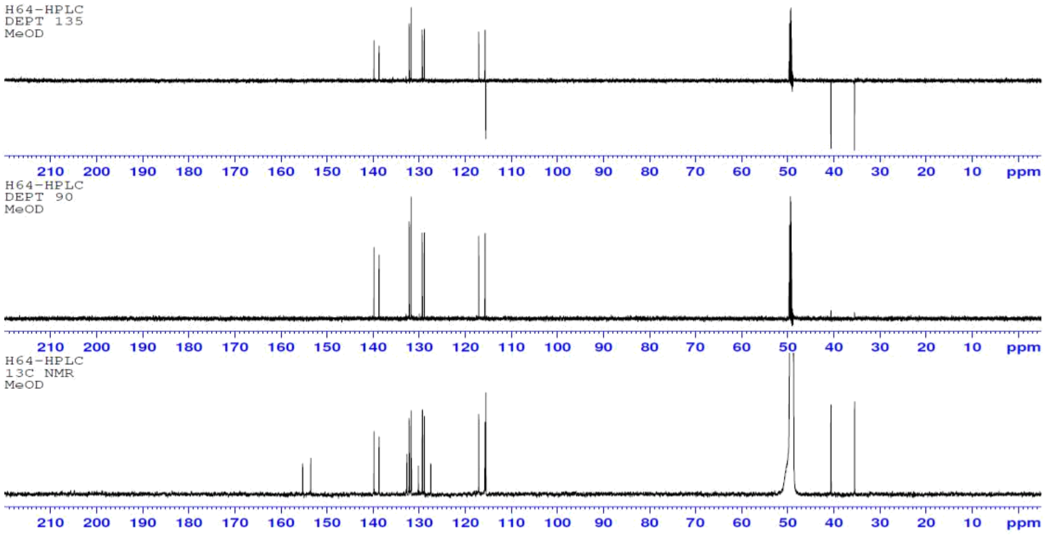

Figure S11. DEPT spectrum of honokiol 3.
